# Dual Stimuli-Responsive Dynamic Covalent Peptide Tags: Toward Sequence-Controlled Release in Tumor-like Microenvironments

**DOI:** 10.1021/jacs.1c06559

**Published:** 2021-10-10

**Authors:** Maksymilian Marek Zegota, Michael Andreas Müller, Bellinda Lantzberg, Gönül Kizilsavas, Jaime A. S. Coelho, Pierpaolo Moscariello, María Martínez-Negro, Svenja Morsbach, Pedro M. P. Gois, Manfred Wagner, David Y. W. Ng, Seah Ling Kuan, Tanja Weil

**Affiliations:** † Max Planck Institute for Polymer Research, Ackermannweg 10, 55128 Mainz, Germany; ‡ Institute of Inorganic Chemistry I, Ulm University, Albert-Einstein-Allee 11, 89081 Ulm, Germany; § Centro de Química Estrutural, Faculty of Sciences, University of Lisbon, Campo Grande, 1749-016 Lisbon, Portugal; ∥ Research Institute for Medicines (iMed.ULisboa), Faculty of Pharmacy, University of Lisbon, 1649-003 Lisbon, Portugal

## Abstract

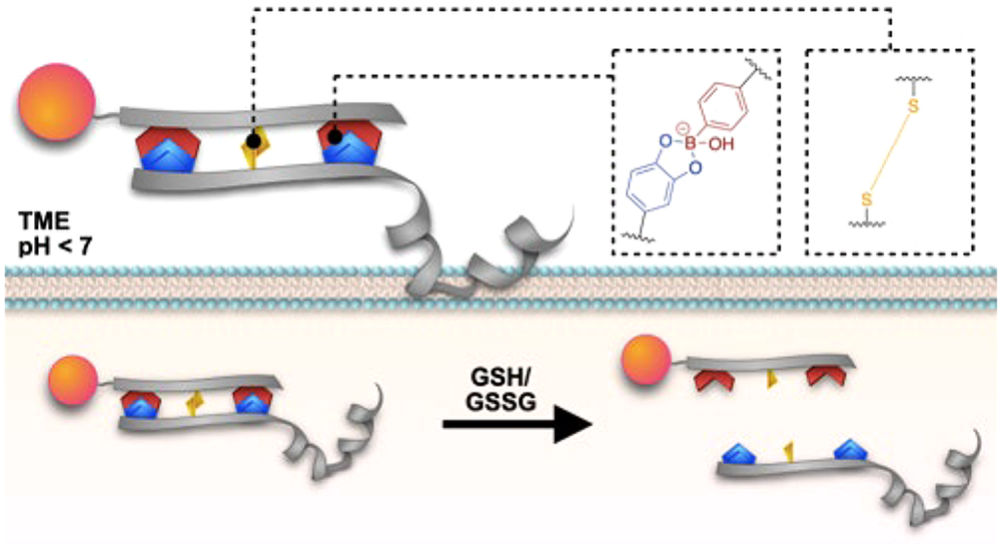

Dynamic covalent chemistry (DCvC) has emerged as a versatile synthetic tool for devising stable, stimuli-responsive linkers or conjugates. The interplay of binding affinity, association and dissociation constants exhibits a strong influence on the selectivity of the reaction, the conversion rate, as well as the stability in aqueous solutions. Nevertheless, dynamic covalent interactions often exhibit fast binding and fast dissociation events or vice versa, affecting their conversion rates or stabilities. To overcome the limitation in linker design, we reported herein dual responsive dynamic covalent peptide tags combining a pH responsive boronate ester with fast association and dissociation rates, and a redox-active disulfide with slow formation and dissociation rate. Precoordination by boronic acid–catechol interaction improves self-sorting and selectivity in disulfide formation into heterodimers. The resulting bis-peptide conjugate exhibited improved complex stability in aqueous solution and acidic tumor-like extracellular microenvironment. Furthermore, the conjugate responds to pH changes within the physiological range as well as to redox conditions found inside cancer cells. Such tags hold great promise, through cooperative effects, for controlling the stability of bioconjugates under dilution in aqueous media, as well as designing intelligent pharmaceutics that react to distinct biological stimuli in cells.

## Introduction

Stimulus-responsive linker chemistry that can differentiate between the biochemical and physical differences manifested by tumor and normal tissues and respond accordingly has emerged as a central tool for the design of smart therapeutics and bioconjugates.^1−3^ For example, systems that exploit the acidic extracellular matrix, acidic intracellular endosomes, elevated temperature, and higher glutathione concentrations found in cancer cells have been designed to trigger drug release selectively.^2,4^ Nevertheless, the field is still fraught with challenges due to unsatisfactory systemic stability or premature drug release of most drug delivery systems.^1,2^ In this regard, a fine balance between stability and reversibility, as well as sensitive response to small changes in the environmental parameters, are highly sought after features when designing linkers for biomedical applications. Dynamic covalent reactions (DCvR) are eminent candidates for new linker design since they are able to provide orthogonality, as well as combining high stability with reversibility at physiologically relevant conditions.^5,6^ Ideally, the DCvRs should possess fast association (high on-rate, *k*_on_) for rapid assembly and efficient conjugation even at lower concentrations, as well as a slow dissociation (low off-rate, *k*_off_) so that the conjugates remain stable upon dilution.

An example of DCvR exhibiting fast *k*_on_ rates is based on phenylboronic acid chemistry with catechol groups, which proceed with fast *k*_on_ rates of about 10^3^ M^–1^ s^–1^,^7,8^ and is comparable to one of the fastest known bioorthogonal reactions, i.e., inverse electron demand Diels–Alder (*k*_on_ > 10^3^ M^–1^ s^–1^).^9^ Moreover, the resultant boronate can achieve release at acidic pH,^8,10^ rendering it attractive for preparing responsive bioconjugates. Nevertheless, fast binding often has its price—fast dissociation and the resultant conjugates usually lack hydrolytic stability.^7,8,11^ For instance, conjugates with salicylhydroxamic acid exhibit high binding affinity (*K*_D_ ∼ 10 μM) but fast dissociation was observed as well (estimated *k*_off_ ∼ 10^–2^ s^–1^).^12,13^ To improve the binding, two peptide strands each containing up to three noncanonical amino acids containing boronic acid and catechol side groups were shown to hybridize and the resultant double stranded peptide conjugates revealed low dissociation constants (the term hybridization is used in accordance with the process of joining two complementary strands of nucleic acids).^14^ However, the limitation with fast dissociation remains unresolved. Conversely, conjugates with dynamic covalent hydrazones/hydrazides, oximes (pH-responsive), and disulfides (redox responsive)^15−17^ showed slow association rates leading to prolonged reaction times, low conjugation yields,^18,19^ and lack of true orthogonality due to homodimerization in the case of disulfides.^20,21^ This can be partially resolved by intramolecular cyclization of disulfides in a single strand using the CXC (cysteine-any-cysteine) motifs but complementarity cannot be achieved.^22,23^ Therefore, achieving fast association in combination with slow dissociation still represents a critical challenge in the design of DCv linkers.^6,24,25^

**Figure 1:**
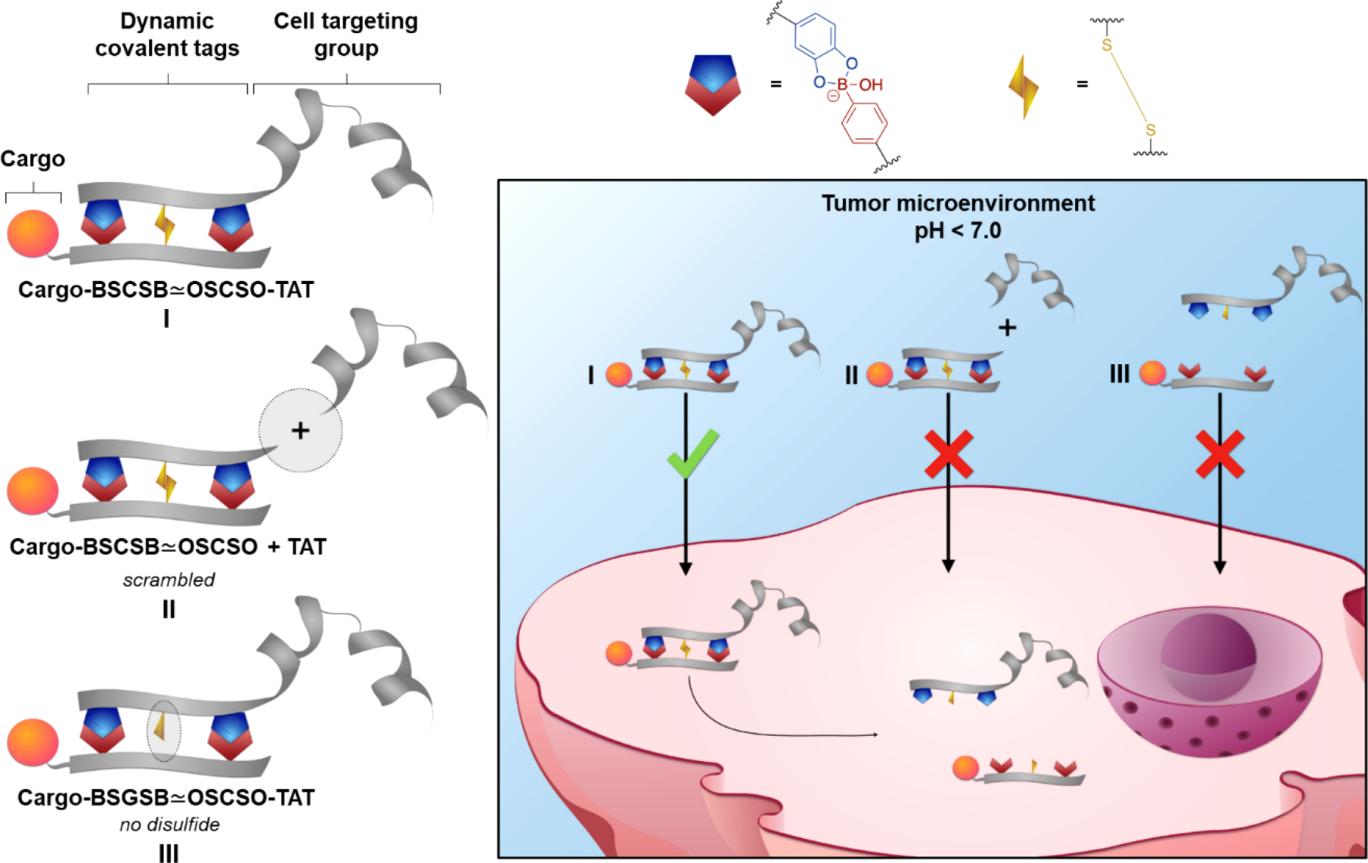
Conceptual overview of multivalent DCvRs based on cooperative boronic acid–catechol and thiol–thiol interaction imparting conjugate stability in the acidic tumor-like extracellular microenvironment as well as controlled release inside tumor cells. Illustrations were made with I–III for comparison.

**Scheme 1:**
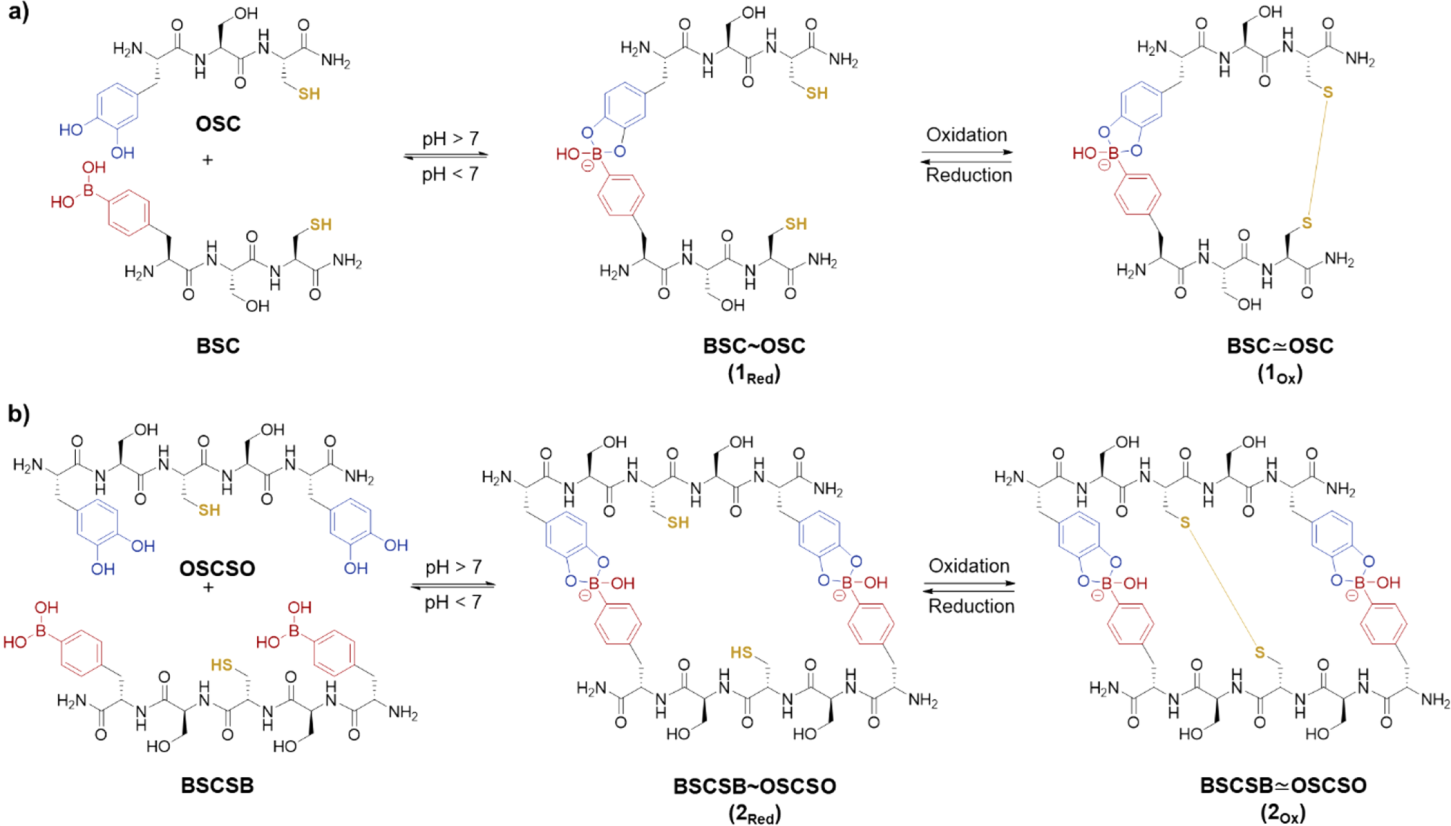
(a) Sequences of the Tripeptide; (b) Sequences of the Pentapeptide All amino acids are represented by single letter code: 4-boronic acid-phenylalanine (B), cysteine (C), serine (S), and 3,4-dihydroxyphenylalanine (O). ∼ denotes dynamic covalent boronate ester formation of B–O in two complementary peptide strands yielding **1_Red_** and **2_Red_**, and ≃ denotes both B–O coordination and oxidation to form boronate ester and disulfide bridge formation leading to the dual stimuli-responsive peptide tags **1_Ox_** and **2_Ox_**.

**Figure 2:**
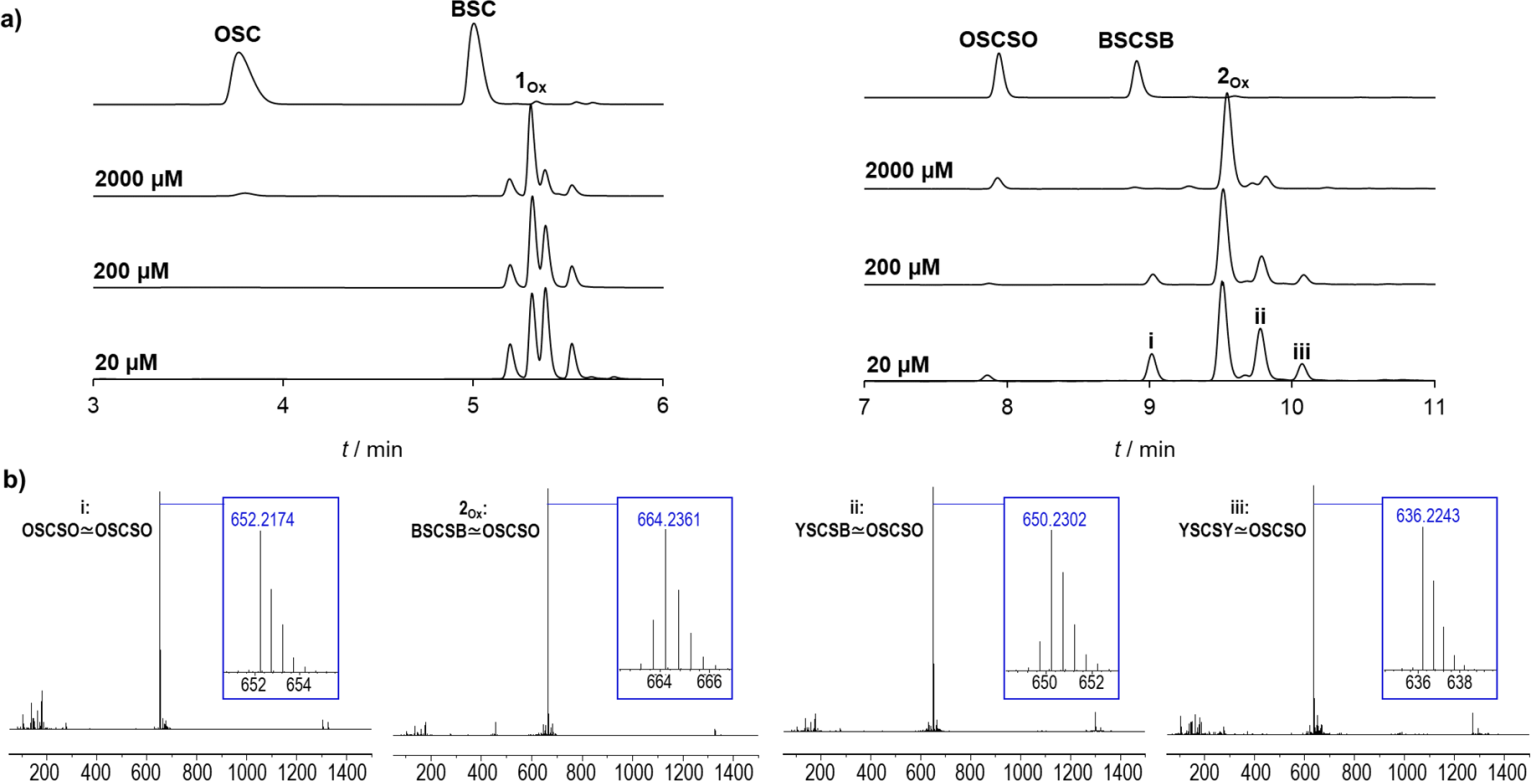
(a) Chromatograms of oxidation reactions using tripeptides (left) and pentapeptides (right) with varying concentrations of binding partners. Reactions were performed in 100 mM phosphate buffer pH = 7.4 by addition of 1.8 equiv of potassium peroxymonosulfate. (b) HR-ESI-MS analysis of purified **2_Ox_** (measured *m*/*z* = 664.2361 [M + 2H]^2+^, calc. *m*/*z* = 664.2343) and side products (i–iii), which were isolated.

New strategies based on two complementary DCvRs compensating each other’s weaknesses are, therefore, imperative to surmount the limitation of individual DCvR.^26^ A DNA-mimetic dynamic covalent system, exploiting pH responsive boronic acid–catechol (fast) and hydrazine–aldehyde (slow) interactions, was devised for selective assembly of molecular ladders and grids from base-4-encoded oligo(peptoid)s.^27^ Nevertheless, a combination of two orthogonal dynamic covalent interactions with two different stimuli on a peptide scaffold, their kinetics, and their stabilities have not been studied yet. In particular, a linker combining boronate esters and disulfides offers (1) fast preassembly due to high *k*_on_ of the boronic acid–catechol interactions, which will convert intermolecular to intramolecular disulfide formation, thereby compensating the slow and unspecific reactivity of thiols to form disulfide bonds; (2) stabilization of the resultant boronate esters due to low *k*_off_ of disulfide (Figure 1, Scheme 1). We demonstrate that short peptide tags containing both cysteine (C) and noncanonical amino acids, 4-boronic acid-phenylalanine (B) and 3,4-dihydroxyphenylalanine (O) residues on the complementary positions exhibit cooperative effects to form new DCv linkers with dissociation features that can be dictated by rationale sequence programming (Figure 2). In addition, targeting peptides such as cell penetrating TAT derived from human immunodeficiency virus can be easily extended on the peptide backbone through solid phase synthesis, and the N-terminal amine can be exploited to incorporate cargoes such as fluorescent dyes to form dual-responsive bioconjugates for drug delivery and bioimaging. Notably, the resultant dual responsive double-stranded bispeptide-linker exhibits stability and, at the same time, reacts to changes in pH or redox conditions, similar to that found in tumor microenvironments. The cooperative DCvR linker strategy presented herein holds immense promise for controlled drug delivery applications.

## Results and Discussion

### Synthesis of Dynamic Covalent Peptide Tags

Four peptide sequences with varying boronic acid, catechol, and cysteine motifs were designed in this study. For clarity, new single letter codes were given for the noncanonical amino acids 4-boronic acid-phenylalanine (B) and 3,4-dihydroxyphenylalanine (or l-DOPA) (O), as depicted in Scheme 1. For solid-phase peptide synthesis (SPPS), the commercially available 4-boronophenylalanine (B) was protected in two steps with Fmoc and pinanediol on the amino and the BA functionality, respectively.^14^

To study the event of multiple boronic acid–catechol interactions, sequences with one or two B and O residues, as well as one cysteine (C) per peptide, were prepared, as shown in Scheme 1a and 1b. The polar amino acid serine (S) was selected as a short spacer that provides sufficient water-solubility with no charges to prevent potential electrostatic repulsion in the spacers in contrast to our previous studies using lysine as a spacer.^14^ All the sequences used in this investigation (BSC, OSC, BSCSB, and OSCSO) and their oxidized heterodimers (**1**_**Ox**_, **2**_**Ox**_) are shown in Scheme 1. The monomeric sequences were synthesized using standard Fmoc-SPPS with *N*,*N*′*-*di-isopropylcarbodiimide/ethyl(hydroxyimino)cyanoacetate (DIC/Oxyma Pure) coupling chemistry, purified using RP-HPLC (>95% purity) and characterized by HR-ESI-MS and MALDI-TOF-MS (SI, Figures S1–S4).

In the following, we will use ∼ to symbolize dynamic covalent boronate ester formation of two complementary peptide strands in their reduced form (**1**_**Red**_ and **2**_**Red**_), whereas ≃ denotes hybridization of complementary peptide strands with boronate ester formation and thiol oxidation to form a disulfide bridge (**1**_**Ox**_ and **2**_**Ox**_).

The hybridization conditions and the influence of B–O precoordination on disulfide formation of complementary tags were first investigated using HPLC. The reaction conditions, i.e., molar ratio of potassium peroxymonosulfate (Oxone), buffer strength, and pH, were optimized (SI, Figure S15a,b). 100 mM phosphate buffer (PB) at pH 7.4 and 1.8 mol equivalents of Oxone were applied for disulfide formation in all subsequent experiments, if not mentioned otherwise (SI, Figure S15a,b). Equimolar stock solutions (4000, 400, or 40 μM in 100 mM phosphate buffer, pH = 7.4) of complementary sequences were mixed in equal volumes and subsequently oxidized with Oxone resulting in final concentrations of 2000, 200, or 20 μM. Due to the sensitivity of the hybridization to pH, it is important to adjust the pH of the solution after dissolution before the oxidation with Oxone. For all studies, hybridized sequences of **1**_**Red**_ and **2**_**Red**_ were prepared by incubation of the individual components at 1 mM, while oxidation with Oxone in situ yield **1**_**Ox**_ and **2**_**Ox**_, as shown in the HPLC-chromatogram (Figure 2a). The reaction mixtures were immediately injected onto RP-HPLC. We observed less side products in the reaction mixture of the pentapeptides BSCSB ≃ OSCSO (**2**_**Ox**_) versus the tripeptides BSC ≃ OSC (**1**_**Ox**_). The incorporation of an additional boronic ester in **2**_**Ox**_ improves chemoselectivity by decreasing the amount of byproducts compared to **1**_**Ox**_ (Figure 2a). Further analysis by LC-MS (SI, Figures S11–S12) confirmed the formation of **1**_**Ox**_ and **2**_**Ox**_, with corresponding masses of 783 (**1**_**Ox**_, calc. *m*/*z* = 782.65) and 1328 (**2**_**Ox**_, calc. *m*/*z* = 1326.97). In addition, the side products were isolated by HPLC and determined by high resolution-ESI-MS (HR-ESI-MS). The homodimer of the catechol sequence, OSCSO ∼ OSCSO (i: measured *m*/*z* = 652.2174 [M + 2H]^2+^; calc. *m*/*z* = 652.2162) was identified, as well as the partially oxidized form of the heterodimer of the boronic acid sequence, YSCSB ∼ OSCSO (ii: measured *m*/*z* = 650.2302 [M + 2H]^2+^; calc. *m*/*z* = 650.2278) and fully deborylated heterodimer YSCSY ∼ OSCSO (iii: measured *m*/*z* = 636.2243 [M + 2H]^2+^; calc. *m*/*z* = 636.2214) (Figure 2b) (SI, Table S1). Additionally we found traces of minor impurities, which were isolated but not characterized due to the quantity (see SI, Figure S15c for full chromatogram).

The benefit of the precoordination on disulfide formation of BSC ≃ OSC (**1**_**Ox**_) and BSCSB ≃ OSCSO (**2**_**Ox**_) is the most pronounced at 2000 μM, but it can be also seen at lower concentrations (200 and 20 μM). In the case of **2**_**Ox**_, fewer side products were formed most likely due to the higher percentage of fraction bound compared to **1**_**Ox**_. Thus, even though the boronic acid moiety is sensitive to oxidation, the amount of side products can be reduced by increasing the monomer concentration or the number of boronic acid–catechol interactions, as seen in the difference between the chromatograms of **1**_**Ox**_ and **2**_**Ox**_. Furthermore, two control sequences which are expected to show no binding were used, BSCSB and YSCSY. The oxidation leads mostly to homodimer with a very small fraction of heterodimer observed (SI, Figure S13), highlighting the importance of the boronic acid–catechol interactions for the selective oxidation to heterodimers. On the basis of the HPLC investigation, which showed conversion rates of approximately 25% for **1**_**Ox**_ and up to 70% for **2**_**Ox**_, peptide complex **2**_**Ox**_ was selected for upscaling and used for all subsequent studies, including NMR studies to assess the structural parameters of the hybridization reaction and the obtained product.

### Structural Analysis of Hybridized Tags by NMR, DFT Calculations, and Circular Dichroism

**Figure 3:**
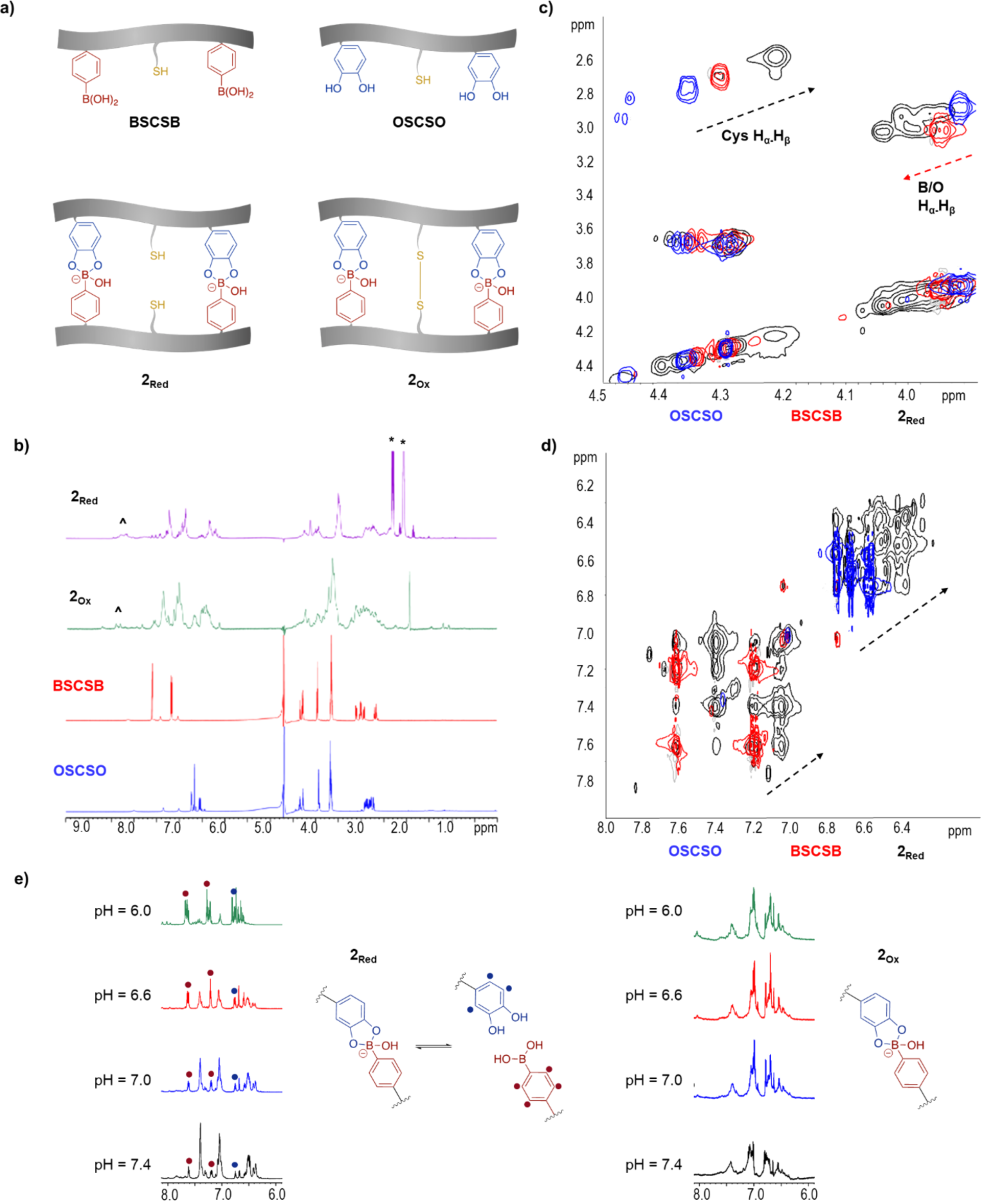
(a) Simplified scheme of the monomers as well as **2_Red_** and **2_Ox_**. (b) ^1^H NMR for peptide monomer and the hybridized double stranded peptide conjugate in reduced (2_Red_) and oxidized (**2_Ox_**) form in 300 mM phosphate buffer, pH 7.4, 10% D2O in H_2_O. * denotes signals due to the reducing reagent TCEP; ∧ are due to minor impurities, presumably due to homodimers and oxidized species. (c,d) ^1^H–^1^H TOCSY for single peptides and **2_Red_** in 300 mM phosphate buffer, pH 7.4, 10% D_2_O in H_2_O (full ^1^H–^1^H TOCSY spectrum and the signal assignment for BSCSB and OSCSO available in the SI, Figures S26 and S27). (e) pH dependent chemical shifts of the aromatic protons in reduced and oxidized form.

The structure and coordination of the hybridized peptide tags, in comparison to the single stranded peptide sequences, as well as oxidized form **2**_**Ox**_ were investigated by a combination of 1- and 2-dimensional NMR spectroscopy (Figure 3). 1D-^1^H NMR shows changes in the entire chemical environment upon boronic acid–catechol (B–O) conjugation in both **2**_**Red**_ and **2**_**Ox**_ (Figure 3b, Figure S24). We observed minor impurities in both spectra (Figure 3b), presumably due to homodimers and overoxidized species, which were also observed in HPLC (Figure 2a). The most affected regions are the H_α_ and the H_β_ protons of the respective cysteines, phenyl boronic acids and catechol (Figure 3c), as well as the aromatic side chain regions, which is clearly shown in the homonuclear total correlation spectroscopy (TOCSY) derived spectra for **2**_**Red**_ (Figure 3d). Upon conjugation, there is a change in the electron density around the protons of both peptides BSCSB and OSCSO, as mirrored in the chemical shift difference in the spectra. With the conformational change upon binding, protons of the amino acids in the proximity of boronic acid and catechol are brought to a position with a deshielding or shielding effect of the “shielding cones”, resulting in a downfield or upfield shift, respectively. The aromatic signals as well as the H_α_ and H_β_ signals of the cysteines show an upfield shift, indicating shielding effects (black arrows, Figure 3c and Figure 3d). On the other hand, a region with an inverse effect, i.e., a downfield shift, was also observed (red arrow, Figure 3c) corresponding to the H_α_ and H_β_ protons of the terminal amino acids in the sequence of each peptide bearing the boronic acid or catechol moieties. Complexation of the complementary peptides (2 mM) was further substantiated by diffusion ordered NMR (DOSY), where slower diffusion of the complex (3.4 × 10^–10^ m^2^ s^–1^) was observed in comparison to the single peptide (3.6 × 10^–10^ m^2^ s^–1^), suggesting an increase of the hydrodynamic radius (SI, Figure S25). The formation of the disulfide was proven by HPLC analysis and discussed in the subsequent section (see Figure 5d).

**Figure 4:**
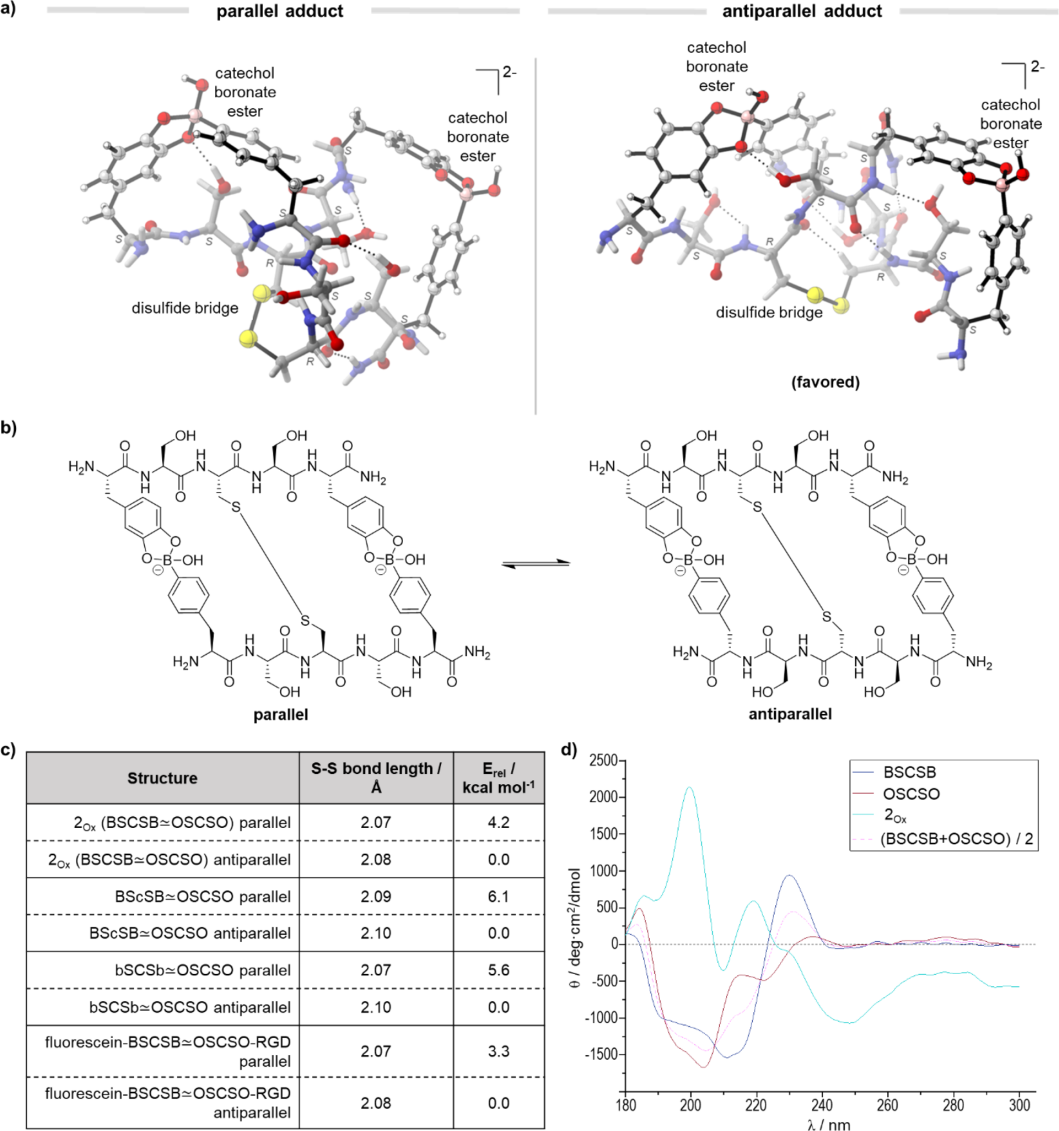
(a) DFT optimized structure of parallel and antiparallel **2_Ox_** at B3LYP/6-31G(d) theory level (color coding: gray, carbon; white, hydrogen; red, oxygen; blue, nitrogen; yellow, sulfur; pink, boron). (b) Chemical structures of **2_Ox_** in the parallel and antiparallel conformation. (c) Table showing calculated S–S bond distance and potential energy from DFT analysis. (d) Circular dichroism of **2_Ox_**, the individual pentapeptides and the average combined spectrum of both pentapeptides.

To gain further structural information on the peptide sequences and the oxidized conjugate **2**_**Ox**_, density functional theory (DFT) calculations were performed at M06-2X/def2-TZVPP/PCM(SMD,water)//B3LYP/6-31G(d) level of theory (Figure 4). The low-lying energy structures of the single peptides (BSCSB and OSCSO) are found to be relatively compact and stabilized by multiple intramolecular hydrogen bonds (SI, Figures S28–S29). Binding entails a linearization of the sequences through formation of the boronate esters, a disulfide bond and intramolecular hydrogen bonds between BSCSB and OSCSO (SI, Figure S30–S31), which heavily influence the chemical environment of the nearby protons, consistent with both the results from ^1^H and multidimensional NMR. The linearization upon binding of the two sequences is further corroborated by CD spectrum, which shows a higher number of distinct bands and higher magnitudes of the bands. These results indicate a higher order structure than that of the single sequences alone (Figure 4d).

Furthermore, calculations suggest that the antiparallel binding (Figure 4b, N-termini on the opposite sides of the conjugate) is favored over the parallel binding (Figure 4b, N-termini on the same side), with a Gibbs free energy difference of 3.5 kcal mol^–1^. To further understand the preference for the antiparallel topology, a distortion/interaction analysis was performed (SI, Figures S38, S40). This analysis reveals stronger overall noncovalent interactions between BSCSB and OSCSO for the antiparallel binding that overpowers the higher degree of distortion of the single peptides in this binding mode.

We further investigated the influence of chirality of different amino acids on the structure. Additional DFT calculations were performed with two additional structures: one where l-Cys in the BSCSB sequence is changed to d-Cys (BScSB) and the other where l-4-borono-phenylalanine is changed to the corresponding d-amino acid (SI, Figures S32–S37). From the DFT calculations, there was only a slight difference in electronic energy (Figure S39), no significant change in the S–S bond length (2.10 versus 2.08 Å) (Figure 4c), and the antiparallel confirmation was preferred in both cases, compared to **2**_**Ox**_ (SI, Table S3). To confirm these findings, the BScSB sequence was synthesized and the hybridized species, BScSB ≃ OSCSO, was detected in LC-MS after oxidation with Oxone (SI, Figure S14).

Finally, to study the influence of bulky substituents at the N-termini of the peptides on the formed hybridized structures, calculations were performed with an additional simplified model analogue comprising of a positively charged RGD sequence on OSCSO and a negatively charged fluorescein dye coupled to the N-termini of the BSCSB sequence (SI, Figures S41–S42). Similarly, the antiparallel binding was determined to be the most favored with a Gibbs free energy difference of 7.5 kcal mol^–1^. This greater difference is attributed to further electrostatic interactions from the positively charged guanidine RGD and the negatively charged dye (SI, Figures S41–S42), suggesting that electronic factors play a role in the arrangement of the peptide sequences.

### NMR Analysis of the Dynamics of Hybridized Tags

Next, the effects of both pH and redox conditions on the hybridized sequences were studied using **2**_**Ox**_ and **2**_**Red**_ utilizing ^1^H NMR spectroscopy. ^1^H NMR spectra of **2**_**Ox**_ are essentially the same in all cases, regardless of the pH range from 6.0 to 7.4 (Figure 3e). The only difference is the appearance of signals between 8.0 and 8.5 ppm, which belong to the amide backbone and is based on the acidity change and thus on the decreased proton exchange rate with the bulk water (Figure 3e). In contrast, under reductive conditions, in the presence of tris(2-carboxyethyl)phosphine (TCEP), the thiol groups are free, and the conjugate is bound only through pH sensitive boronate esters. Decreasing the pH affects the signals originating both from side chains and the backbones (Figure 3e) that split to eventually result in a spectrum resembling simple overlap of single peptides spectra (SI, Figure S24). Nevertheless because of the high concentration, which was about 3 orders of magnitude above the dissociation constant, complete dissociation into the monomers at pH 6 did not occur. Therefore, additional studies were performed under different pH or redox conditions using different methods.

### Dual-Responsiveness of the Dynamic Covalent Tags to pH or Redox Conditions

**Figure 5:**
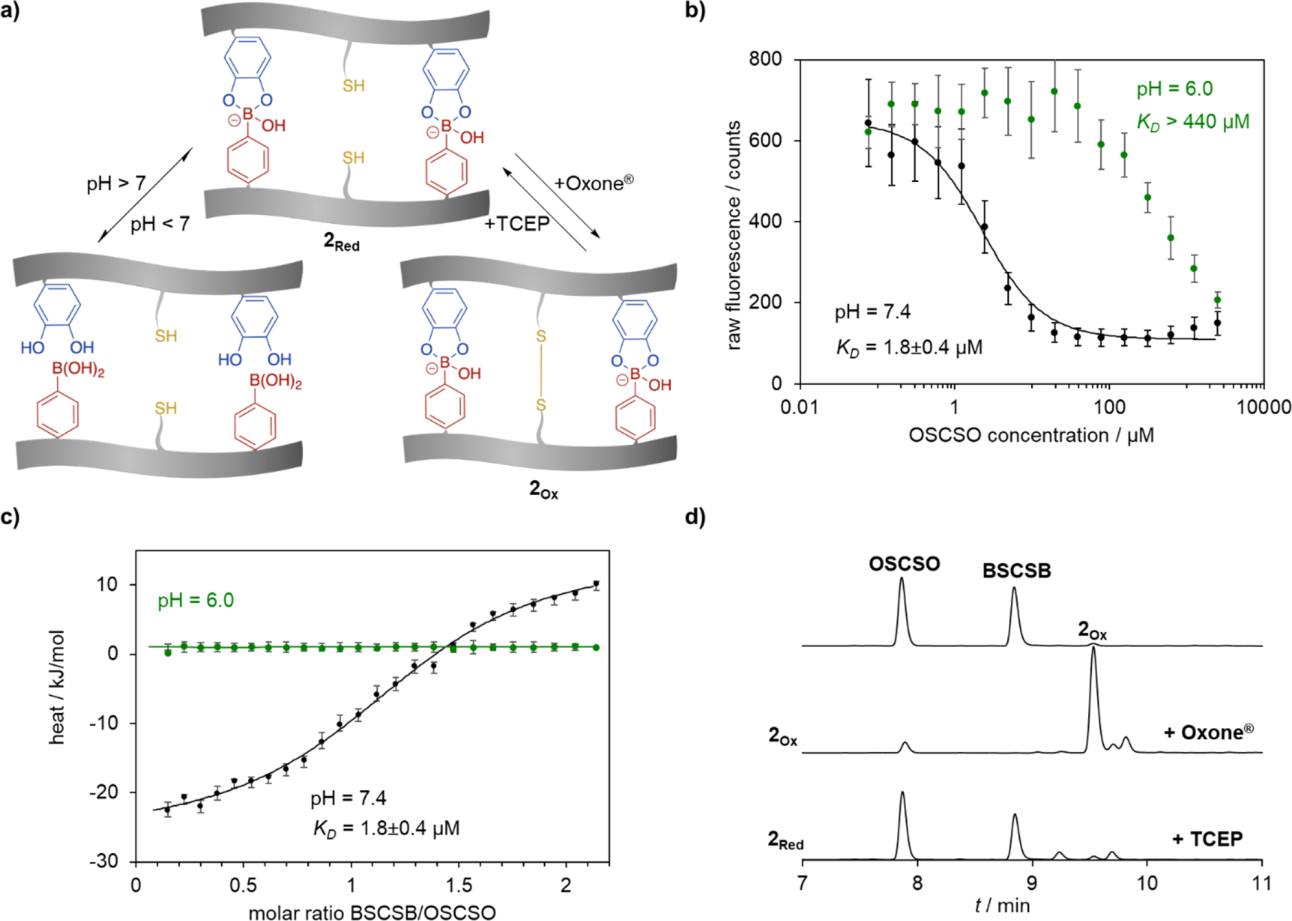
(a) pH- or redox-dependent formation of **2_Ox_** and **2_Red_**. (b) Fluorescence quenching assay conducted in 100 mM phosphate buffer both at pH 7.4 and 6.0 in the presence of TCEP. (c) ITC binding curves showing integrated heats together with an independent binding model fit of **2_Red_** in 100 mM phosphate buffer at pH 7.4 and pH 6.0. (d) HPLC analysis (using mobile phase with 0.1% trifluoroacetic acid) of formation and dissociation of disulfide bond formation under oxidizing or reducing conditions.

The pH-reversible interactions of boronic acid and catechol groups, the formation of disulfides, as well as the responsiveness of the bis-peptide system were further investigated under different conditions (Figure 5). First, the boronic acid condensation with the complementary catechol and the thermodynamic properties were assessed under pH conditions that are relevant to physiological conditions, i.e., pH = 7.4 typical for extracellular environment of normal tissues and pH 6 for acidic intracellular compartments such as endosomes.^28^ Equimolar concentrations of the reacting tags were applied to investigate the thermodynamic parameters associated with the formation of the heterodimer. DyLight488 labeled peptide (DL488-BSCSB) was synthesized by connecting the commercially available NHS-ester of this dye to the N-terminus of the BSCSB sequence to enable a microscale thermophoresis (MST) experiment, in which the dissociation constant could be determined. MST measurements were performed by titrating DL488-BSCSB (2 μM) against the complementary OSCSO peptide binding partner (76 nM to 5 mM). A dissociation constant in the low micromolar range (*K*_D_ = 1.8 ± 0.4 μM, Figure 5b) was obtained for DL488-BSCSB ∼ OSCSO at pH = 7.4, which was 1 order of magnitude lower than the previously reported divalent analogue KOKOK ∼ KBKBK (*K*_D_ = 80.0 ± 7.0 μM)^14^ and 3 orders of magnitude lower than the single phenylboronic acid–catechol interaction (*K*_D_ = 1.2 mM).^14^ Such an improvement could be explained by the lack of electrostatic repulsion due to the presence of the electroneutral serine (at physiological pH) in the sequence. To demonstrate pH responsiveness of the bioconjugate, the pH was adjusted to pH 6, which drastically increased the *K*_D_ to over 440 μM. To assess the binding constant at physiological pH and to understand the type of complex formed, isothermal titration calorimetry (ITC) measurements were performed. The raw data were plotted as heat rate versus time at pH = 6 and 7.4. (SI, Figure S16). The reaction stoichiometry *n* = 1.2 ± 0.1 indicates that primarily 1:1 complexes were formed at pH 7.4. From the independent binding model fit resulting of the integrated heat (Figure 5c), the thermodynamic parameters of interaction were obtained. At pH = 6 the heat of dilution (BSCSB to buffer) was similar to the reaction titration (BSCSB to OSCSO) indicating lack of binding. At pH = 7.4, significant exothermic signals were obtained, yielding a binding constant *K*_D_ = 1.8 ± 0.4 μM, similar to the one previously determined by MST. As a control, BSCSB was titrated to a sequence YSCSY, where l-DOPA was replaced with tyrosine. No binding could be observed, which further strengthens the importance of the l-DOPA/4-borono-phenylalanine motif for binding (SI, Figure S17). It is noteworthy that we are able to maximize reaction mass efficiency by using equimolar concentrations of reagents in these studies, in contrast to literature, where a large excess of one of the reagents is usually applied to improve the conversion.^14^ Importantly, both ITC and MST experiments revealed that about 96% of hybridized species were observed at physiological pH at a concentration of 1 mM. Moreover, these experiments showed that even when the pH is lower than the corresponding p*K*_a_ of the 4-borono-phenylalanine (∼8.9 for 4-methylphenylboronic acid^29^) the binding is sufficient for subsequent investigation in biological media. This observation is consistent with previous findings that the p*K*_a_ is not the sole parameter that affects binding.^14,30^

After precoordination based on the boronic acid–catechol interaction, **2**_**Red**_ can be selectively oxidized to **2**_**Ox**_ by forming a disulfide bridge (Figure 5a). We expect that this secondary S–S dynamic covalent bond locks the conjugate as a heterodimer introducing a reductive environment as a new stimulus for dissociation. Therefore, the formation followed by dissociation of the disulfide was also confirmed by RP-HPLC by subjecting 1 mM solutions of **2**_**Ox**_ and **2**_**Red**_, using the known oxidizing and reducing reagents, Oxone and TCEP respectively, and the peptide monomers were used as standards (Figure 5d). Directly after the addition of Oxone to the **2**_**Red**_ sample solution in PB, we “locked” the bis-peptide to form **2**_**Ox**_ with a retention time higher than any of the single components (Figure 5d). A solution of **2**_**Ox**_ formed by oxidizing **2**_**Red**_ could be reduced subsequently in situ without prior purification by addition of the reducing agent TCEP in slight excess (2.7 equiv), resulting in nearly quantitative hydrolysis of the bis-peptide into the monomeric peptide sequences (BSCSB and OSCSO) under acidic conditions (Figure 5d). This is because conjugate **2**_**Red**_ was formed in the presence of the reducing agent TCEP and further dissociated under acidic conditions of the measurement where the eluent contains additive of 0.1% trifluoroacetic acid (TFA), to afford BSCSB and OSCSO. The two sequences could be further oxidized in situ to form **2**_**Ox**_, albeit with higher amount of side products, due to the excess TCEP used in the previous step (SI, Figure S18). Taken together, our results have shown that the combination of two DCvCs in a single tag has cooperative effects and that the tags react in a pH- or redox-dependent fashion, but full reversibility of the entire system is not achievable under the conditions applied.

### Application of Cooperative Dynamic Covalent Tags under Conditions Mimicking the Tumor Microenvironment

**Figure 6:**
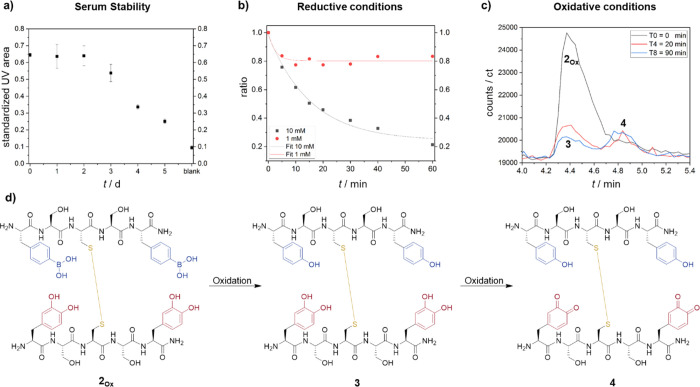
Stability of **2_Ox_** under different conditions. Solutions of **2_Ox_** were incubated in various conditions (a–c) and quantified with selective ion monitoring in LC-MS. (a) 10% fetal calf serum at 37 °C. (b) Reductive conditions with GSH/GSSG in liver cytosol. (c) Oxidative condition with 0.1% hydrogen peroxide. (d) Reaction scheme upon incubation with hydrogen peroxide.

Peptides often reveal low stability in cell media. Moreover, noncovalent interactions provide responsiveness but often cannot allow for sufficient binding affinity and stability in complex cellular environments and under high dilution.^31,32^ Therefore, we investigated the stability of **2**_**Ox**_ in biologically relevant environment. First, serum stability of **2**_**Ox**_ (1 mg mL^–1^, 0.75 mM) was assessed by incubation in 1× phosphate buffer saline (PBS) with 10% fetal calf serum (FCS) at 37 °C and thereafter analyzed using RP-HPLC-MS with Fmoc-Phe-OH as an internal standard (SI, Figure S19). The eluent was acidified with formic acid to exclude the influence of B–O binding. Under these conditions, the conjugate remained stable for up to 2 days and started to decay over the course of 3–5 days (Figure 6a), most likely due to disulfide exchange with cysteine-rich FCS.

Next, the stability of **2**_**Ox**_ in glucose containing solutions were tested since it is known that phenyl boronic acids interact with sugars.^11^**2**_**Ox**_ was incubated in glucose solution (1 g/mL) or glucose depleted DMEM cell media (1 g/mL), which was used in the subsequent cell studies. The analysis was performed for up to 3 days and quantitative analysis was obtained using single ion monitoring (SIM) of *m*/*z* of **2**_**Ox**_ (*m*/*z* = 664, 388) in LC-ESI-MS to improve sensitivity in detection (SI, Figure S20). Fmoc-Phe-OH was used as an internal standard. The results show that **2**_**Ox**_ was determined to be nearly stable in glucose solution for 3 days (SI, Figure S20), suggesting that **2**_**Ox**_ is stable to competitive binding from diols due to stabilization from the disulfide. The amount of **2**_**Ox**_ decreased by around 20% in low glucose DMEM cell culture media after a day of incubation (SI, Figure S20). The decrease is presumably due to the disulfide exchange with free thiols inside the media (10% FCS and cysteine according to manufacturer’s specifications). Nevertheless, **2**_**Ox**_ is sufficiently stable to potential thiol exchange for subsequent biological studies.

Thereafter, we tested the stability under conditions mimicking the tumor microenvironment, where it has been reported that cancer cells possess higher intracellular concentrations of glutathione (GSH/GSSG) in the cytosol, e.g., up to 10:0.25 mM in A549 lung carcinoma cells, compared to concentrations in healthy cells (1:0.025 mM).^4,33^ Such differences in the physiological concentration of GSH have been exploited for controlled release. Thus, the GSH-induced cleavage of 1 mM of **2**_**Ox**_ was probed by incubation in commercially available liver cytosols spiked with physiologically relevant concentrations of GSH:GSSG (1:0.025 and 10:0.25 mM). The analysis was performed for up to 60 min and quantitative analysis was obtained using SIM in LC-MS (**2**_**Ox**_, *m*/*z* = 1328, 664) with Fmoc-Phe-OH as an internal standard (SI, Figure S22). As shown in Figure 6b, the complex was partially reduced when incubated with 1 mM GSH and an equilibrium state was established after approximately 10 min, with 80% of **2**_**Ox**_ unreacted. On the other hand, at a concentration of 10 mM (molar ratio 10:1) the reaction was slightly slower due to longer equilibration time. However, it is apparent that after 60 min, nearly all **2**_**Ox**_ was cleaved to the peptide monomers, BSCSB and OSCSO. Therefore, although the oxidized complex is stable over a longer period in serum (Figure 6a), GSH release could be induced in cytosolic conditions in cancer cells (Figure 6b), which would be important for controlled release as a drug delivery system.

We further determined the stability of the hybridized sequences under oxidative conditions. The oxidized complex, **2**_**Ox**_ was incubated with 0.1% hydrogen peroxide (30 μM). Aliquots were analyzed via LC-MS over different time intervals up to 60 min (SI, Figure S23) using SIM detection. Besides monitoring the decrease in the *m*/*z* of **2**_**Ox**_, SIM also offers the possibility to identify degradation products and allowed us to map the chemical processes involved in the oxidative condition implemented. The SIM profile indicates that no starting material is left after 10 min. We observed complete conversion to compound **3** (Figure 6c,d). After 10 min, the tyrosine compound is further oxidized to the tyrosine and *o*-quinone substance forming compound **4**, which elutes later due to higher hydrophobicity. This result is not surprising, since protected catechols are rather stable against oxidation considering that they are less likely to form oxygen radicals which are crucial in the oxidation process of catechols.^34^ Notably, both sequences remained bound by the disulfide bridge and did not dissociate into the respective single peptide sequences, clearly underlining the stability of the hybridization under tumor-mimicking oxidative condition.

Dynamic covalent tags offer many attractive features for bioconjugate formation as their binding and release could be very useful for the delivery and stimulus-controlled release of drugs. The OSCSO tag was extended with the TAT peptide sequence (YGRKRRQRRR), which is a cell penetrating peptide derived from the human immunodeficiency virus. The TAT sequence has been widely employed for the delivery of cargoes into cells, such as small molecule drugs or proteins. TAT-OSCSO (YGRKRRQRRRS-OSCSO) was synthesized using SPPS and purified by HPLC and identified by MALDI-TOF-MS (*m*/*z* = 2281.1929 [M + H]^+^, calc. 2281.1955 [M + H]^+^), SI, Figure S8). A Dylight488 fluorescence dye (DL488) was conjugated to a BSCSB sequence on the N-terminus as a molecular cargo for transportation by TAT-OSCSO. DL488-BSCSB was purified by RP-HPLC and identified by ESI-MS (SI, Figure S9). The assembly of the dual responsive DL488 ≃ TAT conjugate was performed simply by mixing equal volumes of 2 mM solutions (100 mM phosphate buffer pH 7.4 with 10% DMSO) of targeting unit (TAT-OSCSO) and the cargo (DL488-BSCSB) and addition of Oxone.

**Figure 7:**
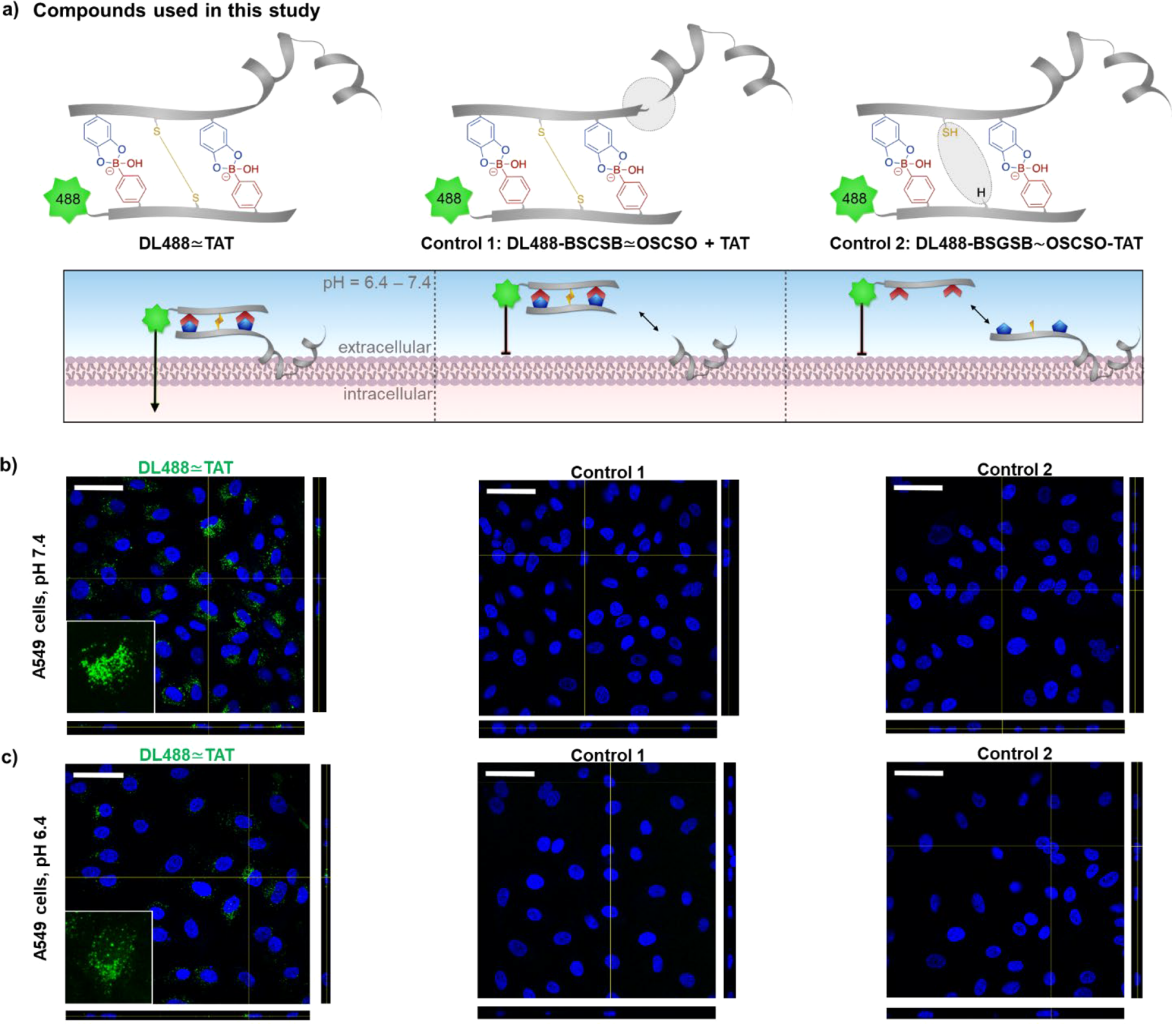
(a) Compounds used in this study and schematic overview. (b,c) Representative confocal orthogonal views of DL488 ≃ TAT (green) uptake in A549 cells both at (b) physiological and (c) acidic pH. Internalization is shown for modified DL488 ≃ TAT and negative controls (1: DL488-BSCSB ≃ OSCSO incubated with free TAT; 2: a conjugate without disulfide bridge (DL488-BSGSB) ∼ (TAT-OSCSO)). All compounds are applied at a concentration of 10 μM. Cell nuclei are shown in blue by DAPI staining. Scale bar = 50 μm.

For cell uptake studies, A549 cell line was selected, which is a model of alveolar Type II pulmonary epithelium.^35^ The uptake was performed under two conditions to test the robustness of the resultant conjugate, which represents the extracellular conditions found in different stages of cancer progression.^36^ Internalization of the conjugate was first studied in standard cell culture media (∼ neutral pH). A solution of DL488 ≃ TAT was applied directly to A549 cells to a final concentration of 10 μM, without any further workup or further purification. As additional controls, (1) a conjugate DL488-BSCSB ≃ OSCSO incubated with free TAT sequence and (2) a conjugate without the disulfide bridge (DL488-BSGSB) ∼ (TAT-OSCSO) where cysteine was replaced by glycine in one of the sequences were both applied at 10 μM (Figure 7a). After 24 h, no uptake was observed in the respective controls while DL488 ≃ TAT showed a significant uptake (Figure 7b). Taken together, the results indicate the immediate assembly of the conjugate and stability both to dilution and incubation in cell medium, as well as TAT-mediated uptake. Next, we proceeded to verify that the peptide tags are robust even under metastatic tumor-like microenvironment, where acidosis is a hallmark. Consequently, the low extracellular pH can result in a more invasive phenotype (Figure 7c), which is more challenging to treat.^28^ First, A549 cancer cells were subjected to acute acidification to derive cells cultured at pH 6.4.^37−39^ Thereafter DL488 ≃ TAT and controls 1 and 2 were applied at 10 μM for 24 h. Notably, the uptake of DL488 ≃ TAT was observed while the negative control 2, with no disulfide formation, was not internalized (Figure 7c). These results suggest that the linkers are stable due to the cooperative effect of two DCv interactions when applied to the acidic extracellular environment. Therefore, the linker chemistry can potentially be adopted for more invasive cancer phenotypes.

## Conclusion

By exploiting the cooperative effect of a multivalent, fast dynamic covalent reaction of boronate esters with that of a slower and more stable dynamic disulfide formation, we have overcome the limitation of each chemistry and showed that a robust system which is dual responsive with tunable binding affinity can be achieved through rational chemical sequence programming. The pH responsive boronic acid–catechol interaction allows precoordination to convert intermolecular to intramolecular thiols of cysteine residues, allowing oxidation to form selectively the heterodimer with significantly reduced reaction time, which offers significant advantage over a single disulfide tag with slower reaction rate and the possibility of disulfide scrambling. The resultant disulfide bond stabilized the conjugate while remaining responsive to a second stimuli-redox environment. Notably, the complementary sequence consisting of two boronate esters is characterized by excellent dissociation constant, outperforming even the binding of single boronic acid with a strong coordinating ligand, i.e., salicylhydroxamate reported previously.^12^ The resultant conjugate is stable under physiological conditions for up to 2 days, to glucose for 3 days, and to intracellular oxidative condition, as well as exhibiting responsive behavior in tumor-like microenvironment (low pH or high GSH concentration). This is important for, e.g., in vivo application where stability in the bloodstream is a major concern, as well as for targeted therapy to avoid unwanted release in normal tissues. Furthermore, very fast conjugation and inertness of the reaction components allows preparation of the conjugate directly before application and use in vitro directly without purification. Remarkably, the construct remains stable in acidic extracellular environment of cancer cells due to the presence of the disulfide bridge, enabling internalization of the cargo. One possible limitation is that the hybridization has to be carried out first at higher concentration (1 mM) and cannot be directly applied for biological applications where low dosage (μM) is required. But this can be resolved by incorporating more B/O in the sequence to increase the binding affinity, as shown in our previous work.^14^ We envisage that the amino acids used in these tags can also be expressed in a protein, thus holding immense promise to become a valuable tool in chemistry and biology to grant intelligent systems through the dynamic and stimuli responsive control over protein assembly or peptide/protein-cargo bioconjugates.

## Supporting Information Available

Structure data (ZIP)
Full experimental procedures and characterization data for new compounds as well as DFT coordinates (PDF)
