# Supplementary material for: Dual Stimuli-Responsive Dynamic Covalent Peptide Tags: Toward Sequence-Controlled Release in Tumor-like Microenvironments
Source: J Am Chem Soc. 2021 Oct 10;143(41):17047–58. doi: 10.1021/jacs.1c06559 (PMC8532147; doi:10.1021/jacs.1c06559)
Supplement: Supplementary file 2 — ja1c06559_si_002.pdf [file ja1c06559_si_002.pdf]

Supplementary Information

Dual stimuli-responsive dynamic covalent peptide tags: Towards sequence-controlled release in tumor-like microenvironments

Maksymilian Marek Zegota, [∇1,2] Michael Andreas Müller,[∇1] Bellinda Lantzberg,[1] Gönül Kizilsavas,[1] Jaime A. S. Coelho,[3] Pierpaolo Moscariello,[1] María Martínez-Negro,[1] Svenja Morsbach,[1] Pedro M. P. Gois,[4] Manfred Wagner,[1] David Y. W. Ng,[1] Seah Ling Kuan,[* 1,2] Tanja Weil[*1,2]

[1]Max Planck Institute for Polymer Research, Ackermannweg 10, 55128 Mainz, Germany

[2]Institute of Inorganic Chemistry I, Ulm University, Albert-Einstein-Allee 11, 89081 Ulm, Germany

[3]Centro de Química Estrutural, Faculty of Sciences, University of Lisbon, Campo Grande, 1749-016 Lisbon, Portugal

[4]Research Institute for Medicines (iMed.ULisboa), Faculty of Pharmacy, University of Lisbon, 1649-003 Lisbon, Portugal

Table of Contents

1. General experimental 2

2. Solid-phase peptide synthesis 2

3. Peptide modification 8

4. Oxidation protocol 9

5. Oxidation optimization 11

6. High performance liquid chromatography (HPLC) experiments and side product identification 12

7. Isothermal titration calorimetry 13

9. Stability studies in serum mimicking media 15

11. Stability in cytosol under oxidative/reductive conditions 17

Stability in liver cytosol 17

Stability under reductive conditions 18

Stability under oxidative conditions 19

12. Fluorescence quenching assay 19

13. ^{1}H NMR experiments: TOCSY, DOSY 19

14. Density functional theory calculations 23

15. Cell uptake studies 39

1. General experimental

Unless otherwise stated, all syntheses were performed without taking precautions to exclude air and moisture. All organic solvents (CH_3CN, CH_2Cl_2, DMF) were obtained from Fisher Scientific and used without further purification (HPLC or peptide grades). H_2O used for the reactions was obtained from the Millipore purification system. Reagents were obtained from Fisher Scientific, Sigma Aldrich and ChemPep Inc. Fmoc-4-boronophenylalanine(pinanediol) was synthesized according to the literature.[12] Reaction progress was monitored by thin layer chromatography (TLC) using Merck 60 F_{254} pre-coated silica gel plates and visualized under ultraviolet lamp (254 nm). Flash column chromatography was carried out using Merck silica gel 60 mesh. Solid-phase peptide synthesis (SPPS) was performed using CEM Liberty Blue peptide synthesizer. High performance liquid chromatography (HPLC) was carried out using Shimadzu HPLC system. NMR spectra were measured on Bruker Avance 850 NMR spectrometer and the chemical shifts (δ) were reported as parts per million (ppm) referenced with respect to the residual solvent peaks. The following abbreviations were used to label the multiplicities: s = singlet, d = doublet, t = triplet, q = quartet, p = pentet, dd = doublet of doublets, dt = doublet of triplets, m = multiplet. MALDI-TOF-MS spectra were acquired on a Bruker Time-of-flight MS rapifleX. HR-ESI-MS was recorded using WATERS SYNAPT G2-Si mass spectrometer. HPLC-ESI-MS analysis was performed on a Shimadzu LC-MS 2020 equipped with an electrospray ionization source and a SPD-20A UV-Vis detector. The absorbance was measured on microplate reader (Tecan Spark 20M). Fluorescence quenching assay was performed using Monolith NT.115.

2. Solid-phase peptide synthesis

Synthesis scale: 0.25 mmol

Deprotection: 20% piperidine in DMF

Activator: 0.25 M DIC (*N*,*N'*-diisopropylcarbodiimide) in DMF

Activator base: 0.5 M Oxyma Pure® (ethyl cyano(hydroxyimino)acetate)

Amino acids: 0.2 M in DMF

Resin: Rink-amide Resin (100-200 mesh), swelled in DMF for 2 h at room temperature

Coupling cycle:

- Standard deprotection (3 mL deprotection solution)

    75 °C (150 W) for 15s

    90 °C (30 W) for 50s

- Wash 3x (2 mL DMF)
- Standard coupling (1.25 mL amino acid, 1 mL activator, 0.5 mL activator base)

    75 °C (150 W) for 15s

90 °C (30 W) for 50s

Final deprotection cycle:

- Standard deprotection (3 mL deprotection solution)

  75 °C (150 W) for 15s

  90 °C (30 W) for 50s

- Wash 3x (2 mL DMF)

Cleavage solution: 4 mL of 95% TFA, 2.5% water, 2.5% TIPS. For deprotection of boronic acid 5 eq. of methylboronic acid was added to the cleavage solution.[13] Cleavage solution was added to the dry resin and stirred overnight at room temperature. Pentapeptides were precipitated by addition of the solution to the cold diethyl ether (-20 °C, 7.5mL per 1mL of cleavage solution) and purified by RP-HPLC. Tripeptides were purified after evaporation of TFA.

HPLC purification

Solvent A: 0.1% TFA in water, solvent B: 0.1% TFA in ACN in a binary gradient

Column 1: Gemini 5 µm, NX-C18, 110 Å (flow rate: 25 mL/min), 150 × 30 mm, column 2: Atlantis T3, 100 Å, 5 µm, 19 mm × 150 mm (flow rate: 10 mL/min)

H-CAT-Ser-Cys-CONH$_2$ (OSC)

HPLC gradient (column 1): 0% B for 1 min, 35% B in 9 min, 100% B in 2 min, 100% B for 1 min. R_t = 8.1 min.

32 mg (62.5 µmol, 25% yield) was obtained from lyophilisation as a TFA salt.

HR-ESI-MS: m/z = 267.0817 $[M\text{-}Cys+H]^+$, 387.1126 $[M+H]^+$, 409.0933 $[M+Na]^+$ (calc. 267.0975 $[M\text{-}Cys+H]^+$, 387.1333 $[M+H]^+$, 409.1152 $[M+Na]^+$, formula: $C_{15}H_{22}N_4O_6S$)

MALDI-TOF-MS: m/z= 387.1135 $[M+H]^+$ (calc. 387.1333 $[M+H]^+$)

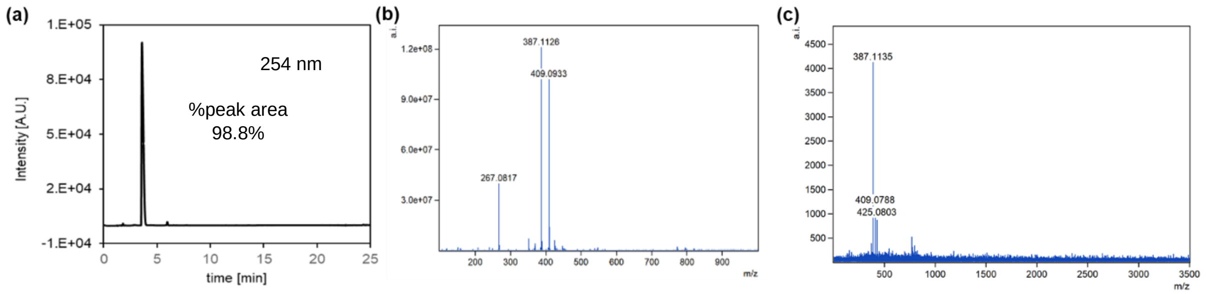

Figure S 1: (a) LC chromatogram and (b) HR-ESI-MS and (c) MALDI-TOF-MS of OSC

H-BPA-Ser-Cys-CONH$_2$ (BSC)

HPLC gradient (column 1): 0% B for 1 min, 35% B in 9 min, 100% B in 2 min, 100% B for 1 min. R_t = 10.0 min.

36 mg (72.5 µmol, 29% yield) was obtained from lyophilisation as a TFA salt.

HR-ESI-MS: m/z = 279.1620 $[M\text{-}Cys+H]^+$, 399.2092 $[M+H]^+$, 421.1940 $[M+Na]^+$ (calc. 279.1147 $[M\text{-}Cys+H]^+$, 399.1504 $[M+H]^+$, 421.1324 $[M+Na]^+$, formula: $C_{15}H_{23}BN_4O_6S$)

MALDI-TOF-MS: m/z= 517.0516 $[M+DHB\text{-}2H_2O+H]^+$ (calc. 517.1559 $[M+DHB\text{-}2H_2O+H]^+$)

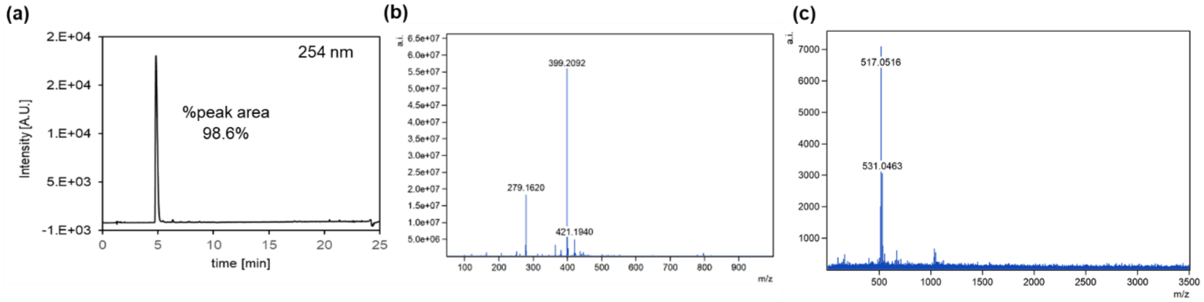

Figure S 2: (a) LC chromatogram and (b) HR-ESI-MS and (c) MALDI-TOF-MS of BSC

H-CAT-Ser-Cys-Ser-CAT-CONH$_2$ (OSCSO)

HPLC gradient (column 1): 0% B for 1 min, 20% B in 9 min, 100% B in 2 min, 100% B for 1 min. R_t = 8.9 min.

100 mg (130.0 µmol, 52% yield) was obtained from lyophilisation as a TFA salt.

HR-ESI-MS: m/z = 346.0846 $[M+H+K]^{2+}$, 653.2164 $[M+H]^+$, 675.21924 $[M+Na]^+$ (calc. 346.0934 $[M+H+K]^{2+}$, 653.2236 $[M+H]^+$, 675.2055 $[M+Na]^+$, formula: $C_{27}H_{36}N_6O_{11}S$)

MALDI-TOF-MS: m/z= 652.9538 $[M+H]^+$, 674.9305 $[M+Na]^+$, 690,9052 $[M+K]^+$ (calc. 653.2236 $[M+H]^+$, 675.2055 $[M+Na]^+$, 691.1794 $[M+K]^+$)

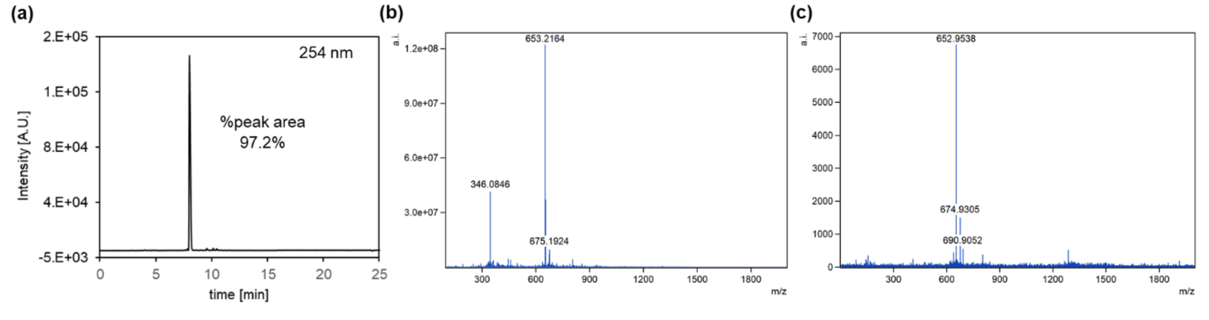

Figure S 3: (a) LC chromatogram and (b) HR-ESI-MS and (c) MALDI-TOF-MS of OSCSO

H-BPA-Ser-Cys-Ser-BPA-CONH$_2$ (BSCSB)

HPLC gradient (column 1): 0% B for 1 min, 20% B in 9 min, 100% B in 2 min, 100% B for 1 min. R_t = 9.1 min.

81 mg (102.5 µmol, 41% yield) was obtained from lyophilisation as a TFA salt.

HR-ESI-MS: m/z = 677.2578 $[M+H]^+$, 699.2272 $[M+Na]^+$ (calc. 677.2578 $[M+H]^+$, 699.2398 $[M+Na]^+$, formula: $C_{27}H_{38}B_2N_6O_{11}S$)

MALDI-TOF-MS: m/z= 913.2117 $[M+2DHB\text{-}4H_2O+H]^+$ (calc. 913.2688 $[M+2DHB\text{-}4H_2O+H]^+$)

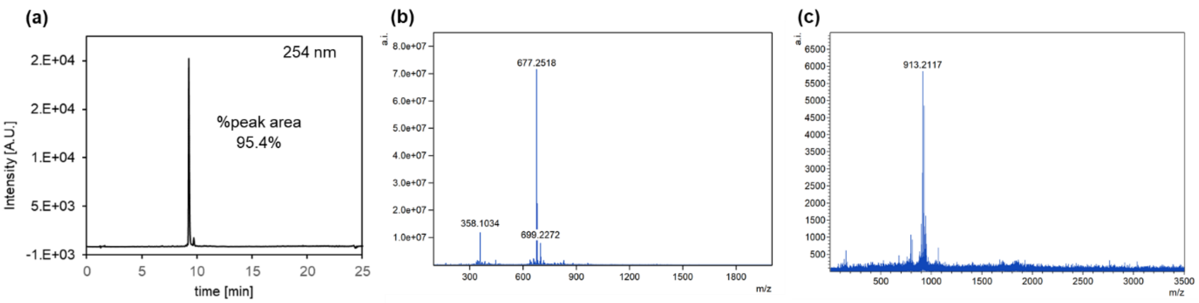

Figure S 4: (a) LC chromatogram and (b) HR-ESI-MS and (c) MALDI-TOF-MS of BSCSB

H-BPA-Ser-Gly-Ser-BPA-$CONH_2$ (BSGSB)

HPLC gradient (column 1): 0% B for 1 min, 20% B in 9 min, 100% B in 2 min, 100% B for 1 min. R_t = 9.1 min.

9.2 mg (16.9 µmol, 34% yield) was obtained from lyophilisation as a TFA salt.

HR-ESI-MS: m/z = 677.2578 $[M+H]^+$, 699.2272 $[M+Na]^+$ (calc. 677.2578 $[M+H]^+$, 699.2398 $[M+Na]^+$, formula: $C_{26}H_{36}B_2N_6O_9S$)

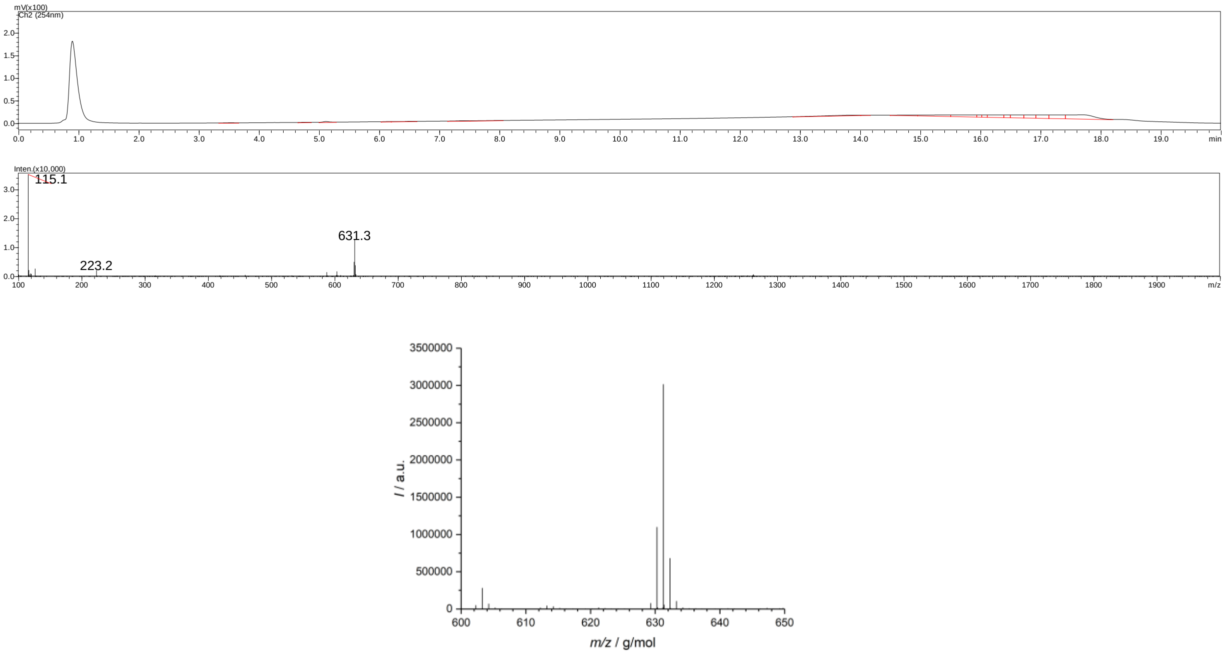

Figure S 5: LC, ESI-MS, HR-ESI of BSGSB.

H-Tyr-Ser-Cys-Ser-Tyr-CONH$_2$ (YSCSY)

HPLC gradient (column 1): 0% B for 1 min, 20% B in 9 min, 100% B in 2 min, 100% B for 1 min. R_t = 9.1 min.

13.6 mg (21.9 µmol, 44% yield) was obtained from lyophilisation as a TFA salt.

HR-ESI-MS: m/z = 677.2578 $[M+H]^+$, 699.2272 $[M+Na]^+$ (calc. 677.2578 $[M+H]^+$, 699.2398 $[M+Na]^+$, formula: $C_{27}H_{36}N_6O_{11}S$)

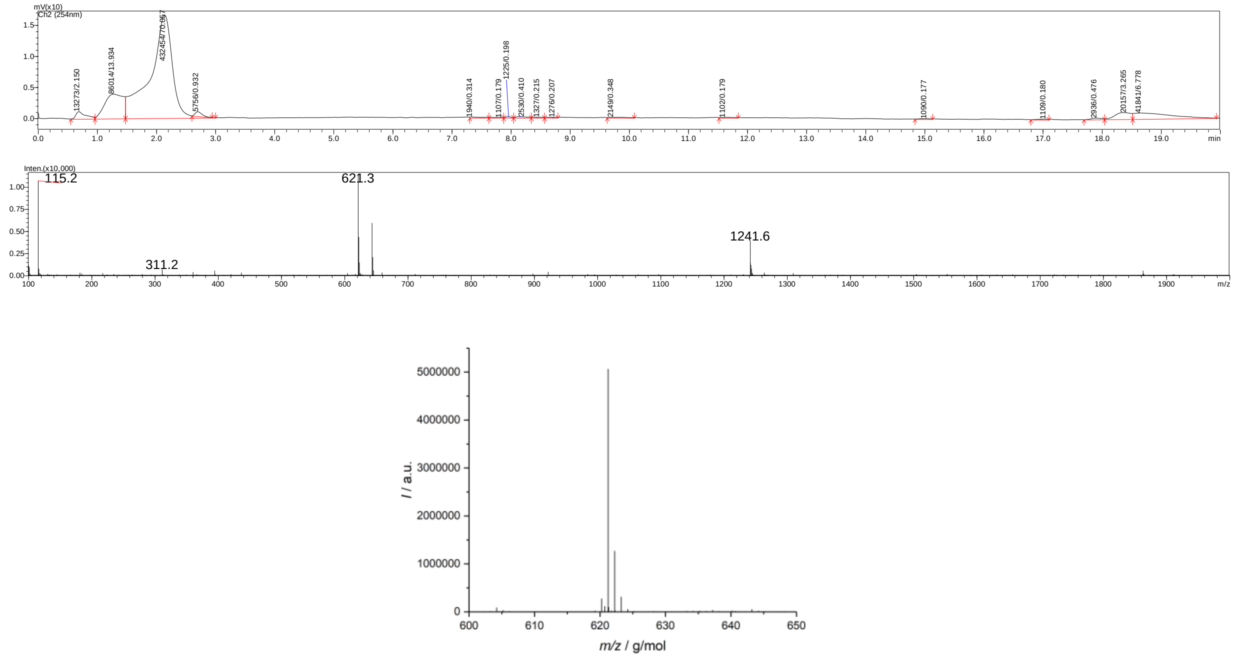

Figure S 6: LC, ESI-MS, HR-ESI of YSCSY.

H-BPA-Ser-D-Cys-Ser-BPA-CONH$_2$ (BScSB)

HPLC gradient (column 1): 0% B for 1 min, 20% B in 9 min, 100% B in 2 min, 100% B for 1 min. R_t = 9.1 min.

4,2 mg (6.2 µmol, 12% yield) was obtained from lyophilisation as a TFA salt. (low yield due to fraction spilling)

ESI-MS: m/z = 677.4 $[M+H]^+$, 699.8 $[M+Na]^+$ (calc. 677.2578 $[M+H]^+$, 699.2398 $[M+Na]^+$, formula: $C_{27}H_{38}B_2N_6O_{11}S$)

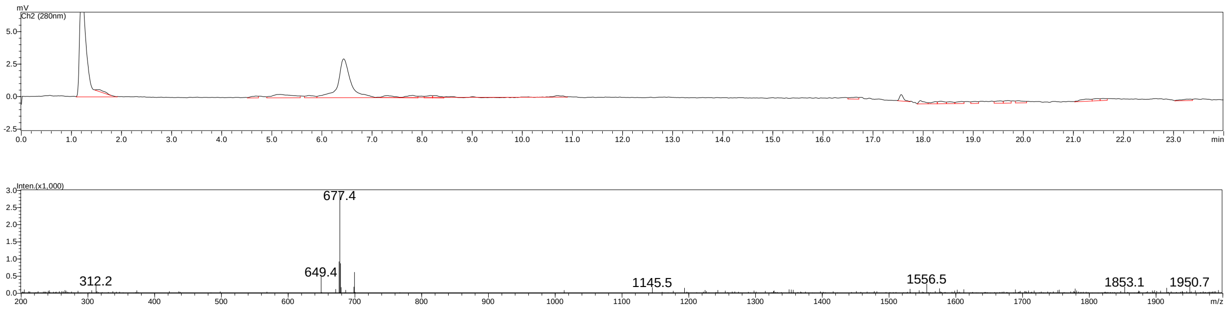

Figure S 7: LC and ESI-MS for BScSB.

H-Tyr-Gly-Arg-Lys-Lys-Arg-Arg-Gln-Arg-Arg-Arg-Ser-Cat-Ser-Cys-Ser-Cat-CONH$_2$ (TAT-OSCSO)

HPLC gradient (column 1): 0%B for 1 min, 40% B in 19 min, 100% B in 2 min, 100% B for 1 min. R_t = 11.9 min.

65 mg (15 µmol, 15% yield) was obtained from lyophilisation as a TFA salt.

ESI-MS: m/z = 457.2256 $[M+5H]^{5+}$, 571.3410 $[M+4H]^{4+}$, 761,3560 $[M+3H]^{3+}$, 1141,7422 $[M+2H]^{2+}$ (calc. 457.0449 $[M+5H]^{5+}$, 571.0543 $[M+4H]^{4+}$, 761,0700 $[M+3H]^{3+}$, 1141,1014 $[M+2H]^{2+}$, formula: $C_{94}H_{157}N_{39}O_{26}S$)

MALDI-TOF-MS: m/z = 2281.1929 $[M+H]^+$ (calc. 2281.1955 $[M+H]^+$)

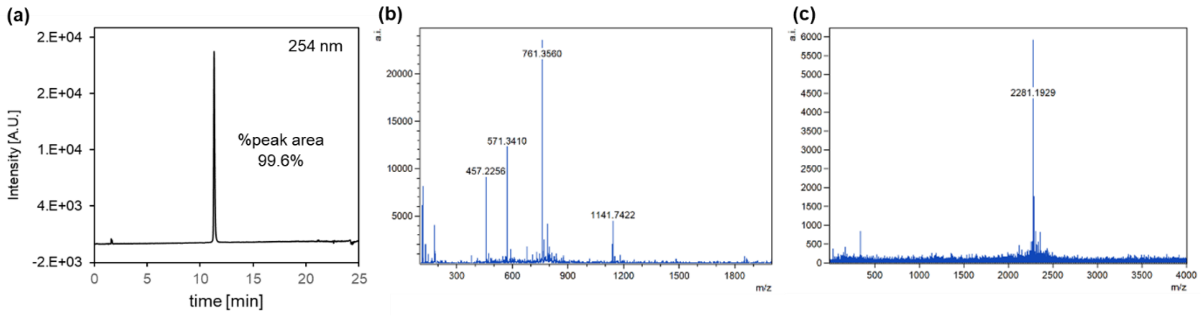

Figure S 8: (a) LC chromatogram and (b) ESI-MS and (c) MALDI-TOF-MS of TAT-OSCSO

3. Peptide modification

Dylight488-BPA-Ser-Cys-Ser-BPA-CONH$_2$ (DL488-BSCSB)

3.2 mg (4.0 µmol, 3 eq.) of BSCSB peptide was dissolved in 1.5 mL of DMF followed by addition of 1 µL (6.0 µmol, 4.5 eq). of triethylamine. Solution was added to 1 mg (1.3 µmol, 1 eq). of the dye NHS ester. Reaction was shaken overnight, DMF was evaporated, residue redissolved in water and purified by RP-HPLC (column 2) using following gradient: 0% B to 35% B in 18 min, 100% B in 1 min, 100% B for 1 min, R_t = 16.1 min. 1.5 mg (1.1 µmol, 85%) of DL488-BSCSB was obtained from lyophilisation as an orange solid.

ESI-MS: m/z = 1314.4 $[M+H]^+$; 655.9 $[M-2H]^{2-}$, 1312.6 $[M-H]^-$ (formula of DyLight488 is not disclosed by the manufacturer)

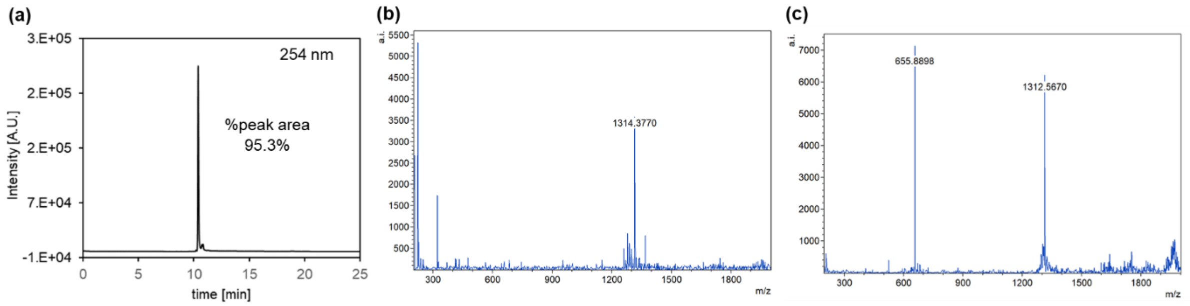

Figure S 9: (a) LC chromatogram and ESI-MS spectra in both b) positive and c) negative ion modes for DL488-BSCSB

Dylight488-BPA-Ser-Gly-Ser-BPA-CONH$_2$ (DL488-BSGSB)

2.5 mg (4.0 µmol, 3 eq.) of BSGSB peptide was dissolved in 1.5 mL of DMF followed by addition of 1 µL (6.0 µmol, 4.5 eq). of triethylamine. Solution was added to 1 mg (1.3 µmol, 1 eq). of the dye NHS ester. Reaction was shaken overnight, DMF was evaporated, residue redissolved in water and purified by RP-HPLC (column 2) using following gradient: 0% B to 35% B in 18 min, 100% B in 1 min, 100% B for 1 min, R_t = 16.1 min. 0.4 mg (0.31 µmol, 24%) of DL488-BSGSB was obtained from lyophilisation as an orange solid.

ESI-MS: m/z = 1311.4 $[M+H]^+$; 654.2 $[M-2H]^{2-}$, 1309.6 $[M-H]^-$ (formula of DyLight488 is not disclosed by the manufacturer)

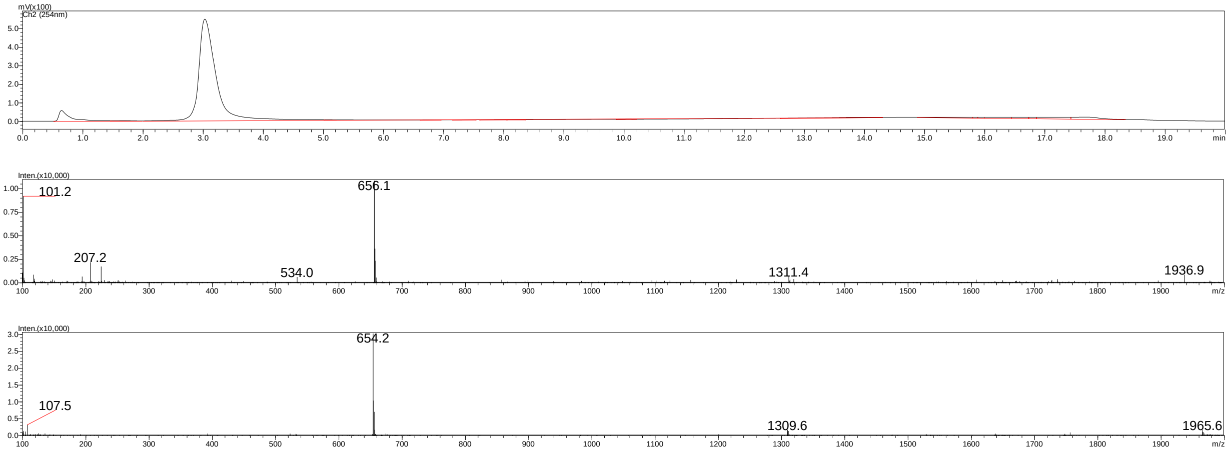

Figure S 10: LC, ESI(+)-MS and ESI(-)-MS for DL488-BSGSB.

4. Oxidation protocol

BSC≃CSO/ BSCSB≃OSCSO /TAT-DL488

50 uL of 2 mM solution of OSC/OSCSO or Tat-OSCSO in 100 mM PB, pH = 7.4 was mixed with 50 uL of 2 mM solution of BSC/BSCSB or DL488-BSCSB in 100 mM PB, pH = 7.4. The mixture was subsequently added rapidly to 2 uL of 90 mM Oxone® solution in 100 mM PB, pH = 7.4 to obtain final Oxone® concentration of 1.8 mM (1.8 eq) and pipetted up and down several times.

BSC≃OSC

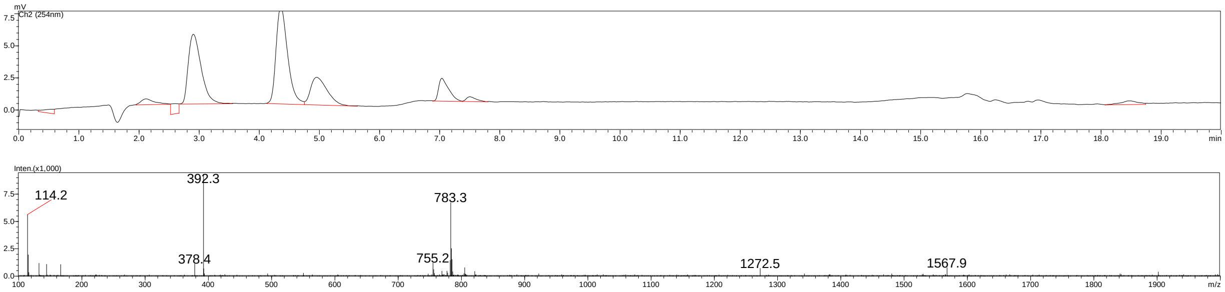

Figure S 11: LC and ESI(+)-MS of the oxidation of BSC≃OSC.

BSCSB≃OSCSO

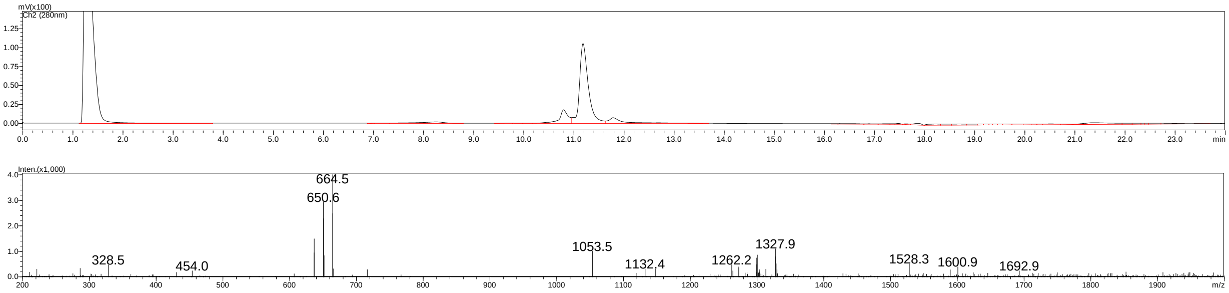

Figure S 12: LC and ESI(+)-MS of the oxidation of BSCSB≃OSCSO.

BSCSB~YSCSY

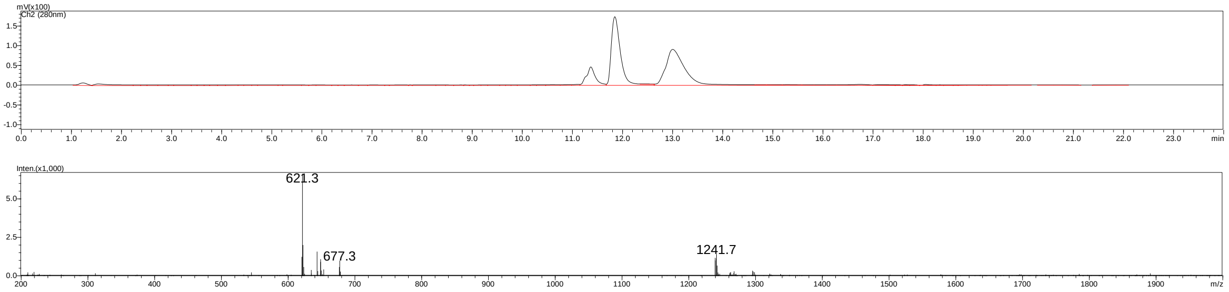

Figure S 13: LC and ESI(+)-MS for the oxidation of BSCSB~YSCSY.

BScSB≃OSCSO

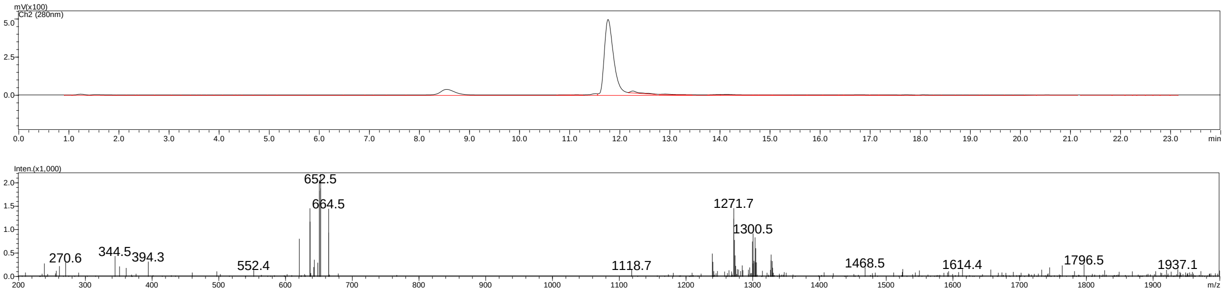

Figure S 14: LC and ESI(+)-MS for the oxidation of BScSB≃OSCSO.

5. Oxidation optimization

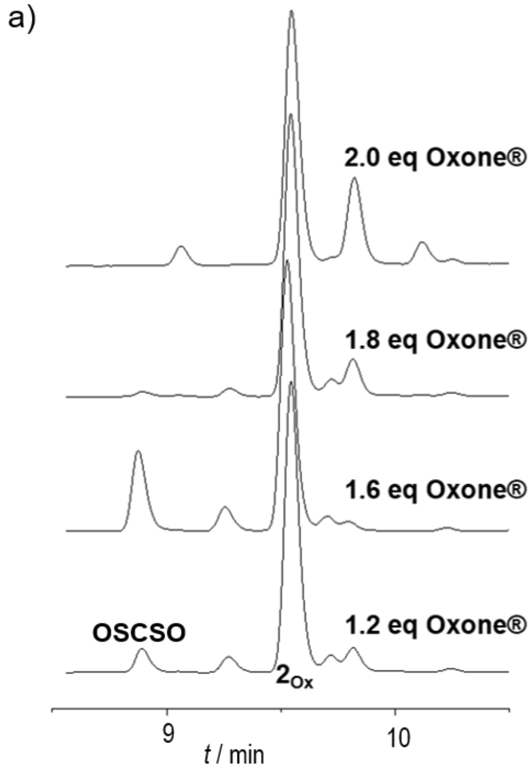

b)

Reaction solvent	Rel. area (2_{Ox})
PB (10 mM, pH 7.4)	0.36704
PB (100 mM, pH 7.4)	0.71609
PB (100 mM, pH 8.0)	0.79208

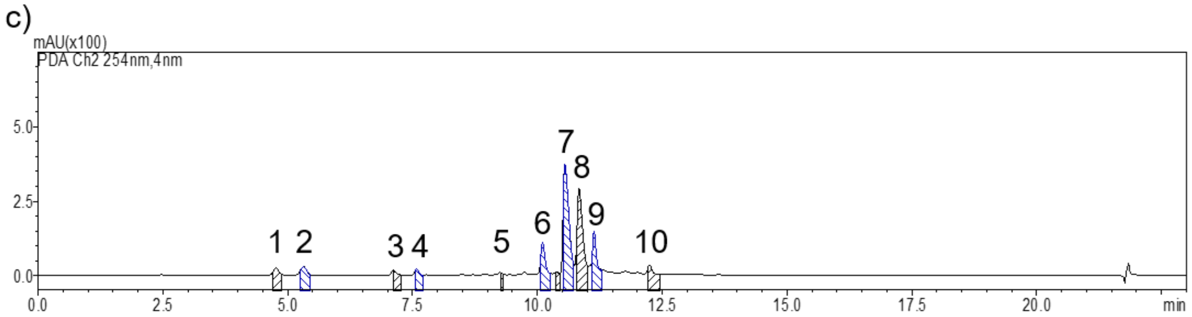

Figure S 15: Oxidation reaction screening of OCSCO ~BSCSB to 2Ox. (a) HPLC chromatograms of oxidation reaction using varying equivalents of potassium peroxymonosulfate (Oxone®). (b) Relative peak areas of 2Ox after 30 min reaction time in varying reaction solvents. Peak areas were normalized to the internal standard. c) Full HPLC-chromatogram of upscaled oxidation protocol with collected fractions (SI, Table S1).

(a) 50 µL of 2 mM solution of OSCSO in 100 mM PB, pH = 7.4 were mixed with 50 µL of 2 mM solution of BSCSB in 100 mM PB, pH = 7.4. The pH of the solution was checked and – if necessary – adjusted to 7.4 using 1M NaOH. The solution was added to 2 µL of Oxone® solution (60, 80, 90 and 100 mM, respectively) in 100 mM PB, pH = 7.4 to obtain a final Oxone® concentration of 1.2, 1.6, 1.8 and 2.0 mM (1.2, 1.6, 1.8 and 2.0 eq). Reaction solutions were diluted (10×, MeOH) and directly injected onto RP-HPLC. Addition of 1.8 eq of Oxone® led to the highest conversion while minimizing side products (Fig. S15).

(b) 47.7 µL of 2.16 mM solution of OSCSO in PB (10 mM, 100 mM pH = 7.4 or 100 mM, pH = 8.0) were mixed with 47.7 µL of 2.16 mM solution of BSCSB in PB (10 mM, 100 mM pH = 7.4 or 100 mM, pH = 8.0) and 1 µL of 50 mM Fmoc-Gly solution in DMSO was added as internal standard. The solution was stirred for 15 min and added to 3.6 µL of 50 mM Oxone® solution in PB (10 mM, 100 mM pH = 7.4 or 100 mM, pH = 8.0) to obtain a final Oxone® concentration of 1.8 mM. 10 µL aliquots of reaction solutions were injected onto RP-HPLC after 5, 30, 60 and 180 min. Higher buffer strength and pH of the reaction solutions led to the highest conversion (Fig. S15).

6. High performance liquid chromatography (HPLC) experiments and side product identification

Column: Agilent ZORBAX Eclipse XDB-C18 (4.6 × 250 mm, 5 µm, 100 Å) thermostated at 40° C

Flow rate: 2 mL/min

Solvents: 0.1% TFA in water (solvent A), 0.1% TFA in acetonitrile (solvent B) in a binary gradient

Gradient for tripeptides: 5% B for 1 min, 100% B in 10 min, 5% B in 1 min, 5% B for 6 min

Gradient for pentapeptides: 5% B for 1 min, 20% B in 9 min, 100% B in 6 min, 100% B for 1.5 min, 5% B in 0.5 min, 5% B for 4 min

All samples were prepared according to oxidation protocol described in point 4, except that vigorous vortexing was applied to the upscaled reaction mixture to induce more side product formation for their identification. All side products visible in the 254 nm UV-trace were collected. Isolated fractions from peaks 5-10 were analyzed using HR-ESI on WATERS SYNAPT G2-Si mass spectrometer. Peaks 1-4 were not analyzed due to the low quantity. Expected masses were calculated as doubly charged species unless otherwise noted based on their chemical formula with MassLynx V4.2.

Table S 1: HR-ESI(+)-MS of isolated side products.

Peak	Identification	Chemical formula	Calc. Mass	Obs. Mass
5	OSCSO	$C_{27}H_{36}N_6O_{11}S$	653.2241 $[M+H]^+$	653.2252
6	OSCSO≃OSCSO	$C_{54}H_{70}N_{12}O_{22}S_2$	652.2162 $[M+2H]^{2+}$	652.2174
7	BSCSB≃OSCSO (2Ox)	$C_{54}H_{72}B_2N_{12}O_{21}S_2$	664.2343 $[M+2H]^{2+}$	664.2361
8	YSCSB≃OSCSO	$C_{54}H_{71}BN_{12}O_{21}S_2$	650.2278 $[M+2H]^{2+}$	650.2302
9	YSCSY≃OSCSO	$C_{54}H_{70}N_{12}O_{20}S_2$	636.2214 $[M+2H]^{2+}$	636.2243
10	YSCSY≃YSCSY	$C_{54}H_{70}N_{12}O_{18}S_2$	620.2264 $[M+2H]^{2+}$	620.2277

7. Isothermal titration calorimetry

The calorimetric measurements were performed using a NanoITC Low Volume (TA Instruments, Eschborn, Germany) with an effective cell volume of 170 µL. In an experiment 50 µL of a BSCSB solution (0.1 mM in a 100 mM phosphate buffer, pH = 7.4 or 6.0) was titrated into a solution of OSCSO (0.016 mM in a 100 mM phosphate buffer, pH = 7.4 or 6.0). The experimental temperature was kept constant at 25 °C. Additionally the same amount of BSCSB solution was titrated into pure water to determine the heat of dilution for reference. As a control another sequence was checked for binding with L-tyrosines instead of L-DOPA and measured as before mentioned. The number and injected volume of the titration steps were the same for all measurements (25 × 2 µL). The spacing between injections was set to 300 s. The integrated reference heats were subtracted from the integrated heats of the adsorption experiments. An independent binding model was employed to fit the adsorption isotherms and obtain the association constant, (K_a), the reaction enthalpy (ΔH), the entropy (ΔS), the Gibbs free energy (ΔG) and reaction stoichiometry (n) (Freire, Mayorga, Straume, 1990; Lewis, Murphy, 2005). The measurements were carried out by triplicate, showing the mean value together the standard deviation for each parameter. Nano Analyze Data Analysis software from TA Instruments was used for the data evaluation of the ITC measurements (Software version 2.5.0) form TA Instruments.

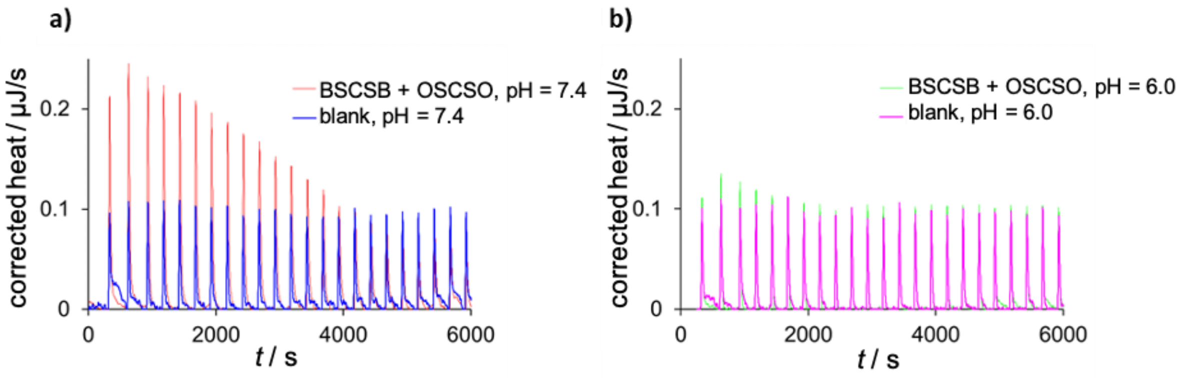

Figure S 16: ITC titrations performed at a) pH = 7.4 and b) 6.0, showing the heat rate signals after base line correction. No heat change different from the dilution (blank) was detected for pH = 6.0.

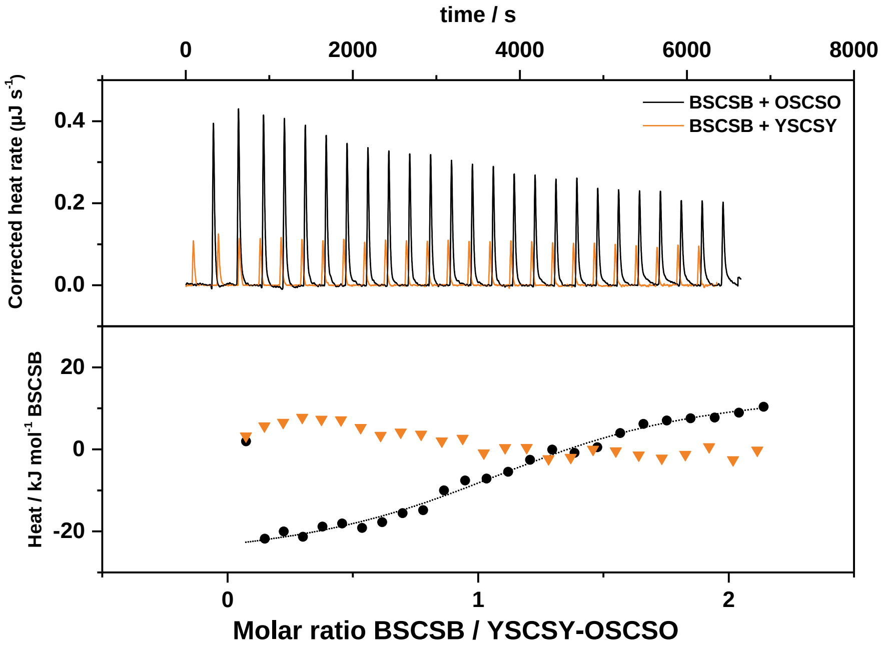

Figure S 17: ITC titrations performed at pH 7.4 with YSCSY and OSCSO as ligands in comparison. Only for OSCSO a significant heat change resulting in a sigmoidal binding curve was detected..

8. Redox cycling experiment

To determine the responsiveness to redox triggers $\mathbf{2_{Red}}$ was subjected to one full redox cycle. First, 75 µL of a 4 mM solution of BSCSB and OSCSO were prepared in 200 mM PB at pH 7.44. The higher buffer strength is to compensate the acidity of Oxone and TCEP. These two solutions were mixed and 10 µL were taken out and diluted with 10 µL MilliQ water and injected into RP-HPLC (Agilent ZORBAX Eclipse XDB-C18 (9.4 × 250 mm, 5 µm, 100 Å) thermostated at 40° C)(Cycle 0). Then 1.8 eq. Oxone were added and gently mixed. Another aliquot was taken out and treated as before (Cycle 1). After that the mixture was treated with 1.7 eq. TCEP and another chromatogram was taken. The reduction was not completed so the batch was split into two and one half was treated with another equivalent of TCEP (Cycle 2). Thereafter, the other half was treated with 2.5 eq. of Oxone and treated as before mentioned (Cycle 3). Furthermore, each cycle was also injected into LCMS to confirm fractions with ESI-MS.

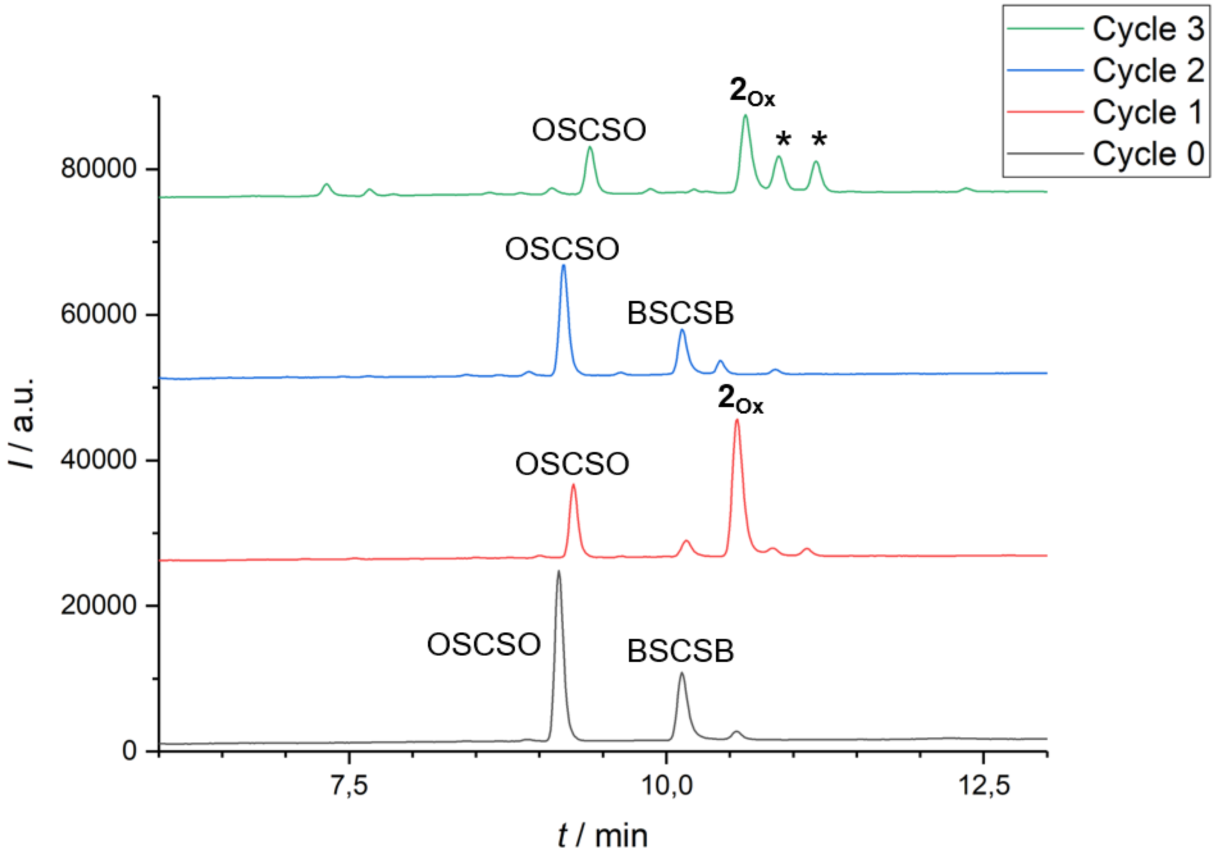

*Figure S 18: Chromatograms of the redox cycling experiment. * denotes side product.*

The first oxidation proceeds with nearly no side products as well as the reduction with 2.7 eq. TCEP to recover starting materials, OSCSO and BSCSB. The second oxidation shows considerable side product formation but we observed formation of $\mathbf{2}_{\mathbf{Ox}}$ again.

9. Stability studies in serum mimicking media

BSCSB≃OSCSO was dissolved in 1 × PBS buffer with 10 % fetal calf serum (FCS) at a concentration of 1 mg/mL and incubated at 37 °C. 10 µL aliquots were taken at intervals of 1 day. Thereafter 90 µL of MeOH containing 20 ppm of Fmoc-Phe as an internal standard (Int. std.) was added to remove large proteins and the solution was centrifuged at 13.0 rpm for 30 min at 0°C. 10 µL of the supernatant was used for HPLC-MS analysis. Experiments were performed in triplicates. Blank consisting of 10 µL 1 × PBS buffer with 10 % fetal calf serum (FCS) treated the same way as the sample was used as a negative control. HPLC-MS analysis was performed on a Shimadzu LC-MS 2020 equipped with an electrospray ionization source and a SPD-20A UV-Vis detector (Shimadzu, Duisburg, Germany).

Column: Kinetex EVO C18 (50 × 2.1 mm, 2.6 µm, 100 Å) thermostated at 40° C

Flow rate: 0.4 mL/min

Solvents: 0.1% FA in water (solvent A), 0.1% FA in acetonitrile (solvent B) in a binary gradient

Gradient: 5%B for 1 min, 70% B in 11 min, 95% B in 1 min, 95% B for 1 min, 5% B in 0.01 min, 5% B for 4 min

Decay of BSCSB≃OSCSO was observed simultaneously by UV detection at 214 nm and MS. The amount of BSCSB≃OSCSO in each sample was determined as a ratio of the integration of the UV peak of BSCSB≃OSCSO to the internal standard. The data were plotted as mean ± SEM.

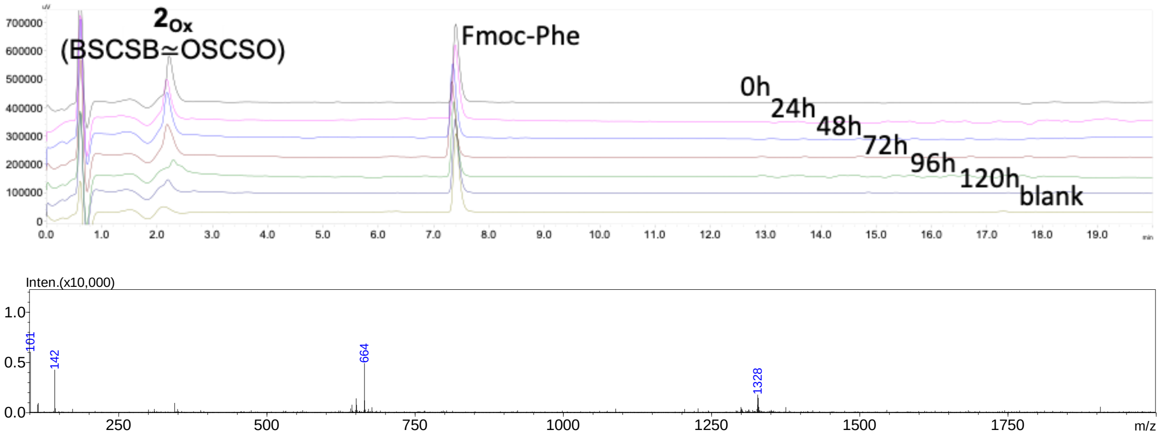

Figure S 19: Chromatograms for BSCSB≃OSCSO aliquoted daily for 5 days and a negative control together with internal standard (Fmoc-Phe). LC-ESI-MS: m/z = 664 [M+2H]$^{2+}$, 1328 [M+H]$^{+}$ (calc. 664.23 [M+2H]$^{2+}$, 1327.46 [M+H]$^{+}$, chemical formula: $C_{54}H_{72}B_2N_{12}O_{22}S_2$).

10. Stability in glucose solution and low glucose cell media

2_{Ox} (1 mM) was incubated at 20 °C in glucose solution (GS, 1 g/ml) and DMEM low glucose cell media (LGM) with 10% FCS. 20 µL Aliquots were taken out and diluted with 80 µL MeOH with 20 ppm Fmoc-Phe and 0.1% FA, then spinned down and 80 µL of the clear supernatant solution was taken out and half was injected into HPLC-MS. The time points were taken each day.

HPLC-MS analysis was performed on a Shimadzu LC-MS 2020 equipped with an electrospray ionization source and a SPD-20A UV-Vis detector (Shimadzu, Duisburg, Germany).

Column: Kinetex EVO C18 (200 × 2.1 mm, 2.6 µm, 100 Å) thermostated at 40 °C

Flow rate: 0.4 mL/min

Solvents: 0.1% FA in water (solvent A), 0.1% FA in acetonitrile (solvent B) in a binary gradient

Gradient: 5% B for 1 min, 20% B in 11 min, 95% B in 1 min, 95% B for 1 min, 5% B in 0.01 min, 5% B for 4 min

SIM (m/z): 1328, 664, 388

The UV (280 nm) signals were integrated and normalized against the Fmoc-Phe standard as well as the SIM for 664 against the SIM of 388. The relative area was then plotted against the incubation time.

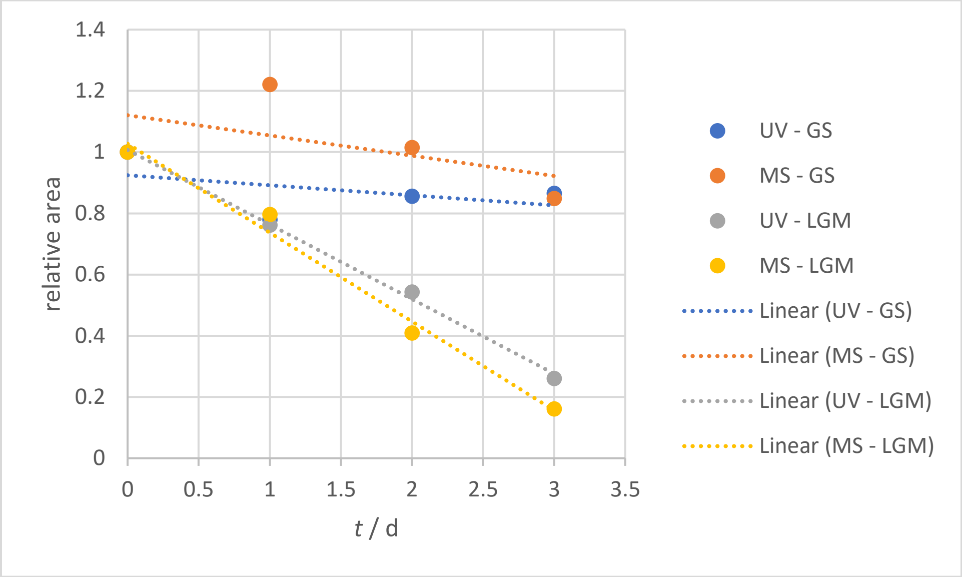

Figure S 20: Stability of $\mathbf{2}_{Ox}$ *in glucose solution (GS) and low glucose cell media (LGM) detected by UV-Vis and ESI-MS.*

$\mathbf{2}_{Ox}$ remains nearly stable in glucose solution whereas it dissociates gradually in cell media, which was expected due to free thiols in the media (FCS and cysteine).

11. Stability in cytosol under oxidative/reductive conditions

Stability in liver cytosol

A solution of BSCSB≃OSCSO of 1 mg ml^{-1} was prepared in 150 µL liver cytosol in an Eppendorf TM tube. The solution was incubated at 37 °C while gentle shaking was applied. In well defined time intervals 10 µL sample was taken out and precipitated 90 µL ice cold methanol with 20 ppm Fmoc-Phe as an internal standard. Then the sample was centrifuged with a tabletop microcentrifuge at maximum speed (4000 rpm). 80 µL of the clear supernatant was transferred into an LC-MS vial and 25 µL were injected into the LC-MS. To deal with the delay caused by the measuring time of 20 min, the samples were stored in liquid nitrogen and directly thawed before injection.

HPLC-MS analysis was performed on a Shimadzu LC-MS 2020 equipped with an electrospray ionization source and a SPD-20A UV-Vis detector (Shimadzu, Duisburg, Germany).

Column: Kinetex EVO C18 (50 × 2.1 mm, 2.6 µm, 100 Å) thermostated at 40 °C

Flow rate: 0.4 mL/min

Solvents: 0.1% FA in water (solvent A), 0.1% FA in acetonitrile (solvent B) in a binary gradient

Gradient: 5% B for 1 min, 70% B in 11 min, 95% B in 1 min, 95% B for 1 min, 5% B in 0.01 min, 5% B for 4 min

SIM (m/z): 1328, 664

Time points	Time [min]
T_0	0
T_1	20
T_2	40
T_3	60
T_4	120
T_5	240
T_6	480

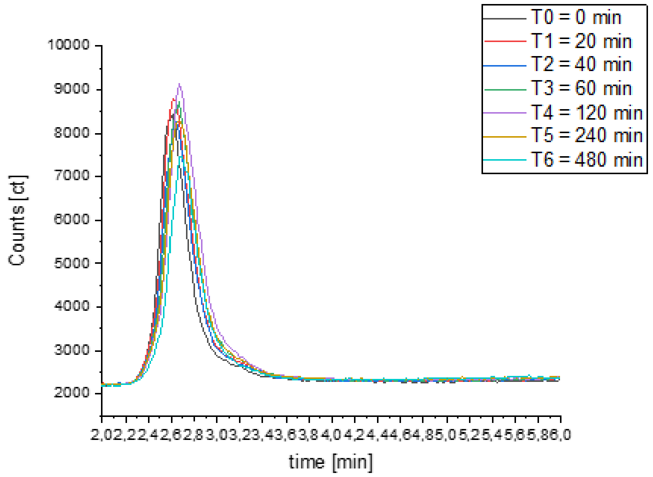

Figure S 21: Time points and SIM(664)-trace for the stability in liver cytosol.

Stability under reductive conditions

The liver cytosol was spiked with glutathione and the corresponding disulfide in a ratio of 40:1. Two different concentrations were used to mimic cancer cell and healthy cell conditions with 10 mM and 1 mM respectively. With this medium a 1 mM solution of BSCSB≃OSCSO was prepared and treated as mentioned above with different time points shown in Fig S10.

Time points	Time [min]
T_0	0
T_1	5
T_2	10
T_3	15
T_4	20
T_5	30
T_6	40
T_7	60

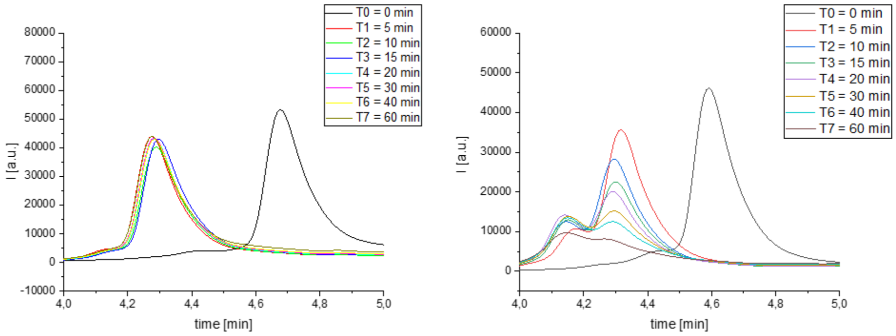

Figure S 22: Time points and SIM(664)-traces for the stability in liver cytosol under reductive conditions for 1 mM and 10mM GSH concentrations respectively.

Stability under oxidative conditions

A 1 mM solution of BSCSB≃OSCSO in ammonium bicarbonate containing 0.1% hydrogen peroxide (30 μM) was prepared and treated as before mentioned.

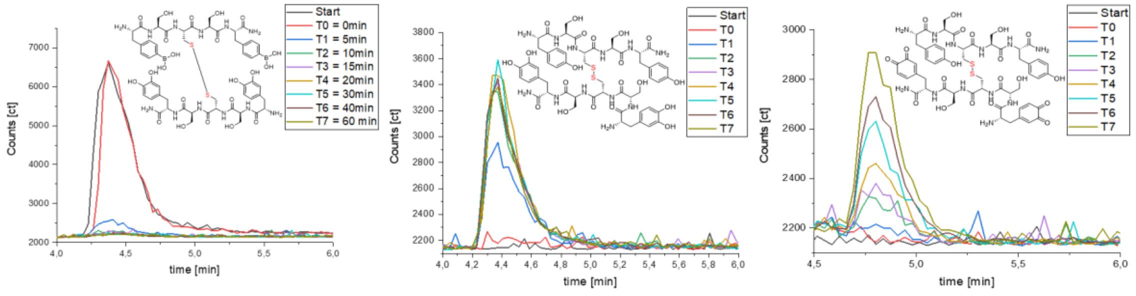

Figure S 23: SIM(664)-trace, SIM(1271)-trace, SIM(1267)-trace.

12. Fluorescence quenching assay

Four stock solutions were prepared: 5 mM OSCSO, 2 μM DL488-BSCSB in a phosphate buffer (100 mM, pH = 6.0, 2.5 mM TCEP) and 5 mM OSCSO, 2 μM DL488-BSCSB in a phosphate buffer (100 mM, pH = 7.4, 2.5 mM TCEP). OSCSO peptide was diluted in a series (1:1) 15 times to obtain concentration ranging from 5 mM down to 76 nM. Each of 16 samples from the series were mixed 1:1 with DL488-BSCSB stock solution and loaded into standard treated capillaries. FQA was measured immediately.

The procedure was repeated 3 times both for pH 6.0 and 7.4. Each point on the graph represents mean average of normalized fluorescence for one concentration from the series and error bars represent standard errors of the mean.

Binding of DL488-BSCSB by OSCSO was detected with K_d = 1.8 ± 0.4 μM at pH = 7.4. Decreasing pH to 6.0 increases binding constant by over 2 orders of magnitude proving pH responsiveness of the binding.

13. ^{1}H-NMR experiments: TOCSY, DOSY

For the NMR analysis, all peptide samples used for this study were dissolved in 600μl 90% H_2O/10% D_2O yielding a typical concentration of 2 mM.

The NMR spectra were recorded on a Bruker AVANCE III 850 MHz spectrometer with a 5 mm triple resonance TXI 1H/13C/15N probe equipped with a z-gradient. All spectra were measured at 298 K. The spectra were analyzed using Bruker TopSpin 3.6.1.

For ^{1}H NMR measurements 512 scans were collected at a 17006.803 Hz (20 ppm) spectral width together with a recycling delay of 2 s and water suppression using watergate W5 pulse sequence with gradients using

double echo and an acquisition time of 1 s (Liu, Mao et al. 1998). The ^{1}H 90° pulse was calibrated for each sample individually and was used for further measurements.

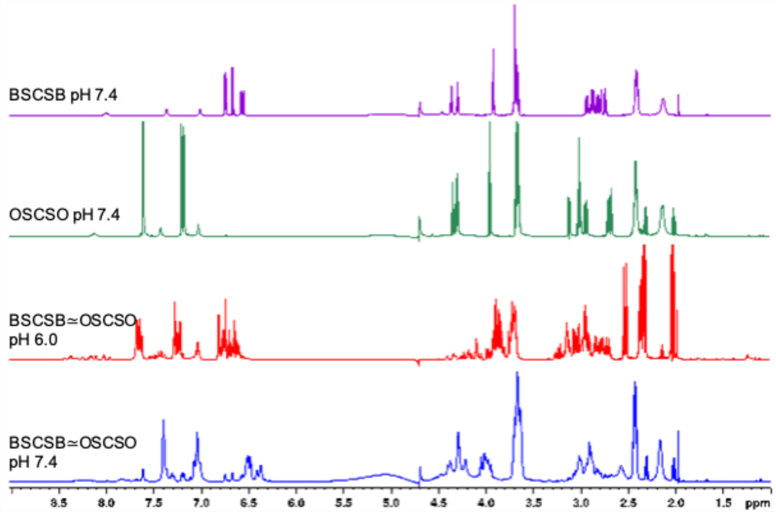

Figure S 24: Comparison of ^{1}H NMR of single tags at pH 7.4 and BSCSB≃OSCSO both at pH 6.0 and 7.4.

DOSY (Diffusion Ordered Spectroscopy) experiments were executed using stimulated echo using bipolar gradient pulses for diffusion using 1 spoil gradient water suppression using 3-9-19 pulse sequence with gradients. In these experiments we used 32 gradient steps from 2 % to 100 % with a total gradient strength of 53 G/cm. The diffusion time d20 was optimized to 80 ms and the gradient length p30 was kept at 1.6 ms.

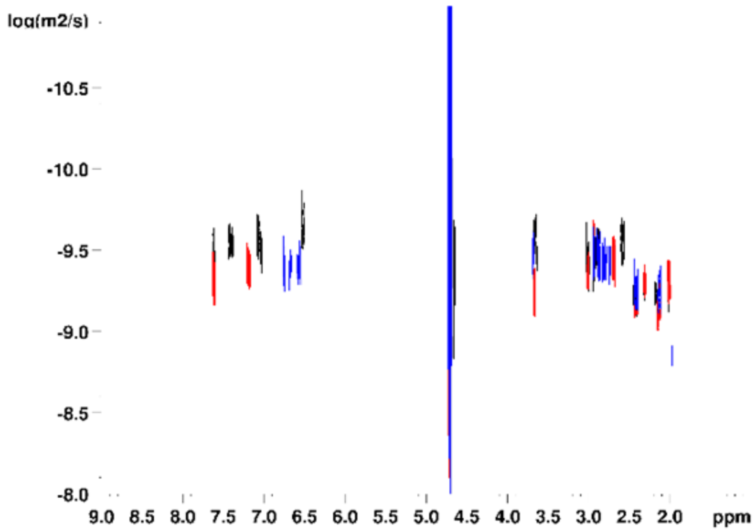

Figure S 25: DOSY for single peptides (red – BSCSB, blue – OSCSO) and BSCSB~OSCSO (black) at pH 7.4.

TOCSY (Braunschweiler and Ernst, 1983; Bax and Davis, 1985) spectra were collected using 256 t1 increments and spectral widths of 12000 Hz in both dimensions. Acquisition times were set to 1.38 s, relaxation delays were 2 s and spin-lock (MLEV-17) mixing times were 80 ms. The spin-lock pulse width (90°) was 20 μs and the trim pulses were set to 2.5 ms.

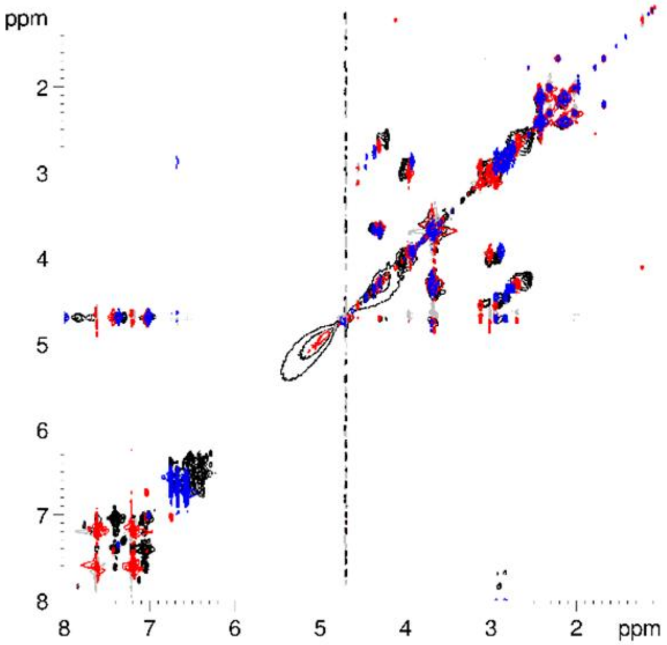

*Figure S 26: ^{1}H-^{1}H TOCSY spectra for the single peptides **BSCSB** in red, **OSCSO** in blue, and the **BSCSB≃OSCSO** conjugate in black in phosphate buffer, with 1 mM TCEP at pH 7.4.*

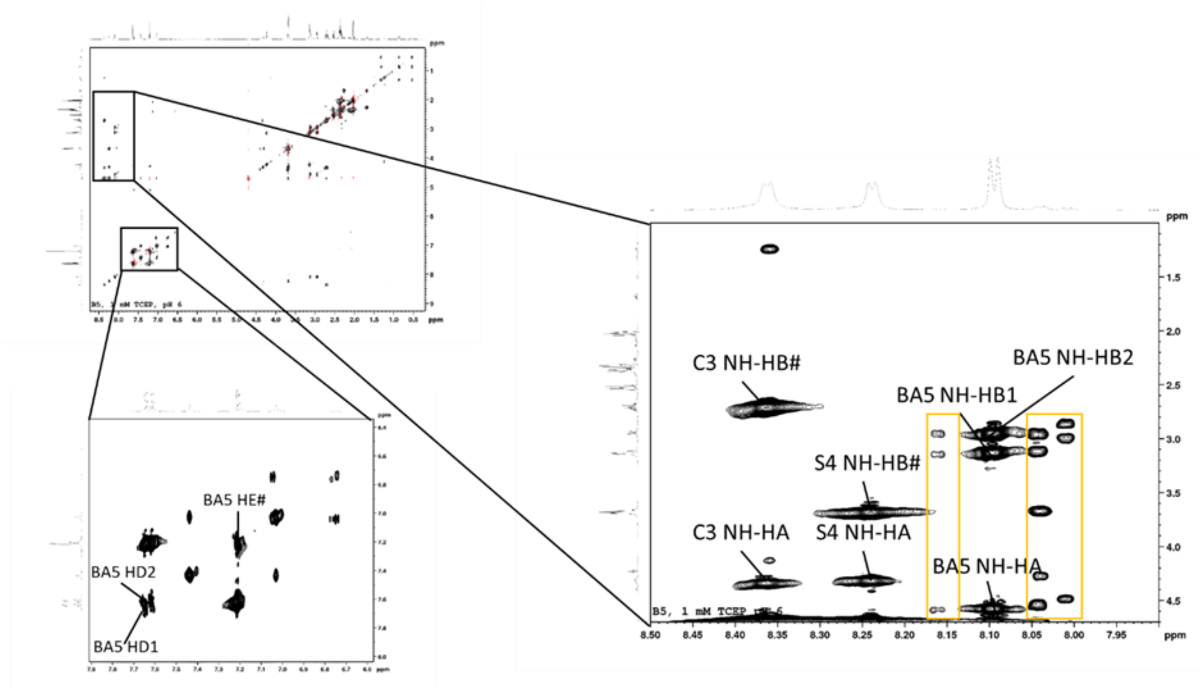

Figure S 27: [1]H-^{1}H TOCSY signal assignment for BSCSB

Table S 2: Proton assignments for a) BSCSB and b) OSCSO sequences.

a)

Group	Atom	Nuc	Shift
BA1	HA	1H	4.229
BA1	HB#	1H	3.144
S2	HA	1H	4.394
S2	HB#	1H	3.693
C3	HA	1H	4.335
C3	HB#	1H	2.711
C3	HN	1H	8.362
S4	HA	1H	4.314
S4	HB#	1H	3.680
S4	HN	1H	8.239
BA5	HA	1H	4.572
BA5	HB1	1H	3.129
BA5	HB2	1H	2.946
BA5	HD1	1H	7.643
BA5	HD2	1H	7.613
BA5	HE#	1H	7.214
BA5	HN	1H	8.099

b)

Group	Atom	Nuc	Shift
CAT1	HA	1H	4.162
CAT1	HB#	1H	2.99
S2	HA	1H	4.417
S2	HB#	1H	3.699
C3	HA	1H	4.4
C3	HB#	1H	2.79
C3	HN	1H	8.377
S4	HA	1H	4.328
S4	HB#	1H	3.712
S4	HN	1H	8.323
CAT5	HA	1H	4.473
CAT5	HB1	1H	2.945
CAT5	HB2	1H	2.831
CAT5	HD1	1H	6.784
CAT5	HD2	1H	6.745
CAT5	HE	1H	6.595
CAT5	HN	1H	7.954

14. Density functional theory calculations

Computational details

Conformational analysis with molecular mechanics (MM) was performed with Schrödinger Maestro 11.8[1], using the force field OPLS3e[2], solvent water, and the default method built in the program.

Density functional theory (DFT) calculations were performed using the Gaussian 09 software package[3] and structural representations were generated with *CYLview*[4]. All the geometry optimizations were carried out using the standard B3LYP functional and the valence double-zeta 6-31G(d) basis set. All of the optimized geometries were verified by frequency computations as minima (zero imaginary frequencies). Single-point energy calculations on the optimized geometries were then evaluated using the hybrid meta-GGA functional M06-2X developed by Truhlar and co-workers[5] and the valence triple-zeta Def2-TZVPP basis set, with solvent effects (water) calculated by means of the Polarizable Continuum Model (PCM) initially devised by Tomasi and co-workers,[6-9] with radii and non-electrostatic terms of the SMD solvation model, developed by Truhler and co-workers.[10] Thermal corrections were calculated from the unscaled vibrational frequencies at the B3LYP/6-31G(d) level on the optimized geometries. Entropic contributions to the reported free energies were calculated from partition functions evaluated using Truhlar's quasiharmonic (qh) approximation.[11] This method uses the same approximations as the usual harmonic one except that all vibrational frequencies lower than 100 cm^{-1} are set equal to 100 cm^{-1}.

Distortion/interaction analysis was performed by single-point energy calculations on the separated single peptides at the M06-2X/def2-TZVPP/PCM(SMD,water) level of theory. The structures of the separated peptides were obtained using the following protocol: 1) separation of the peptides of **2_{Ox} parallel** and **2_{Ox} antiparallel**, 2) addition of missing atoms (O and H) to complete the structures, 3) optimization at B3LYP/6-31G(d) level freezing all the original atoms i.e., except the atoms added in 2).

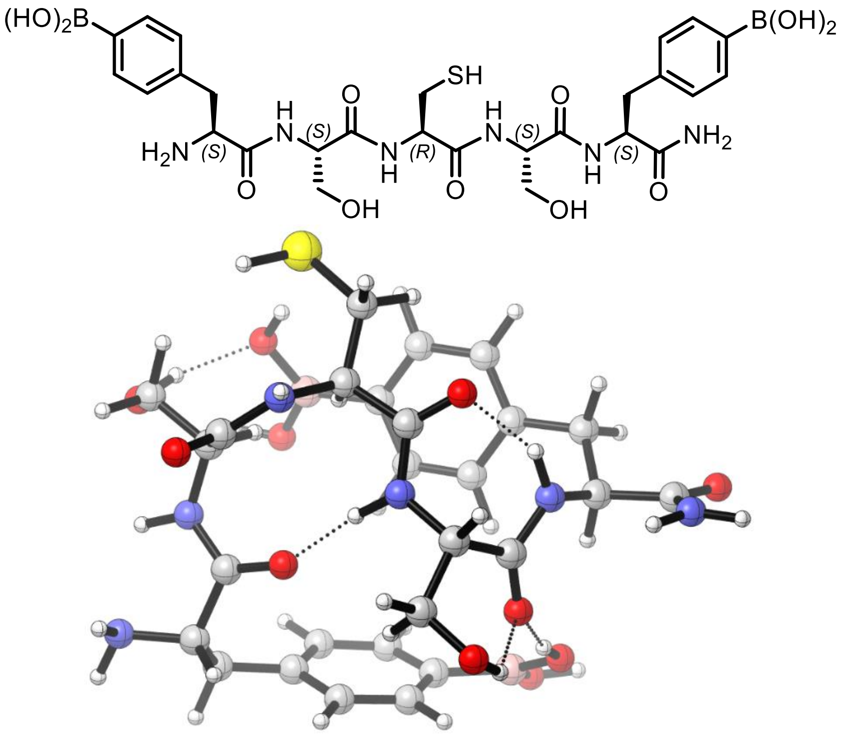

Figure S 28: Skeletal structure and low-lying conformer of BSCSB.

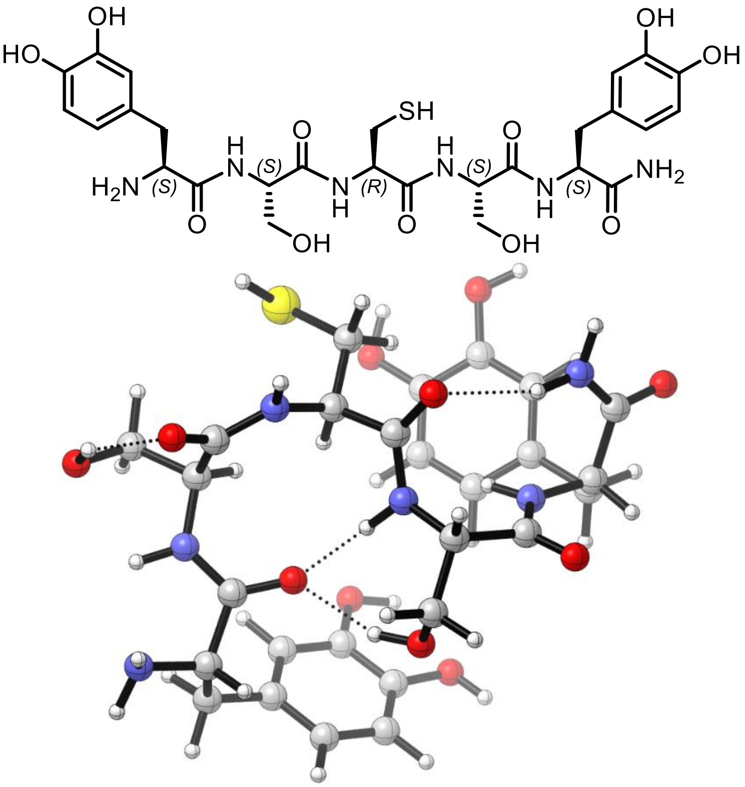

Figure S 29: Skeletal structure and low-lying conformer of OSCSO.

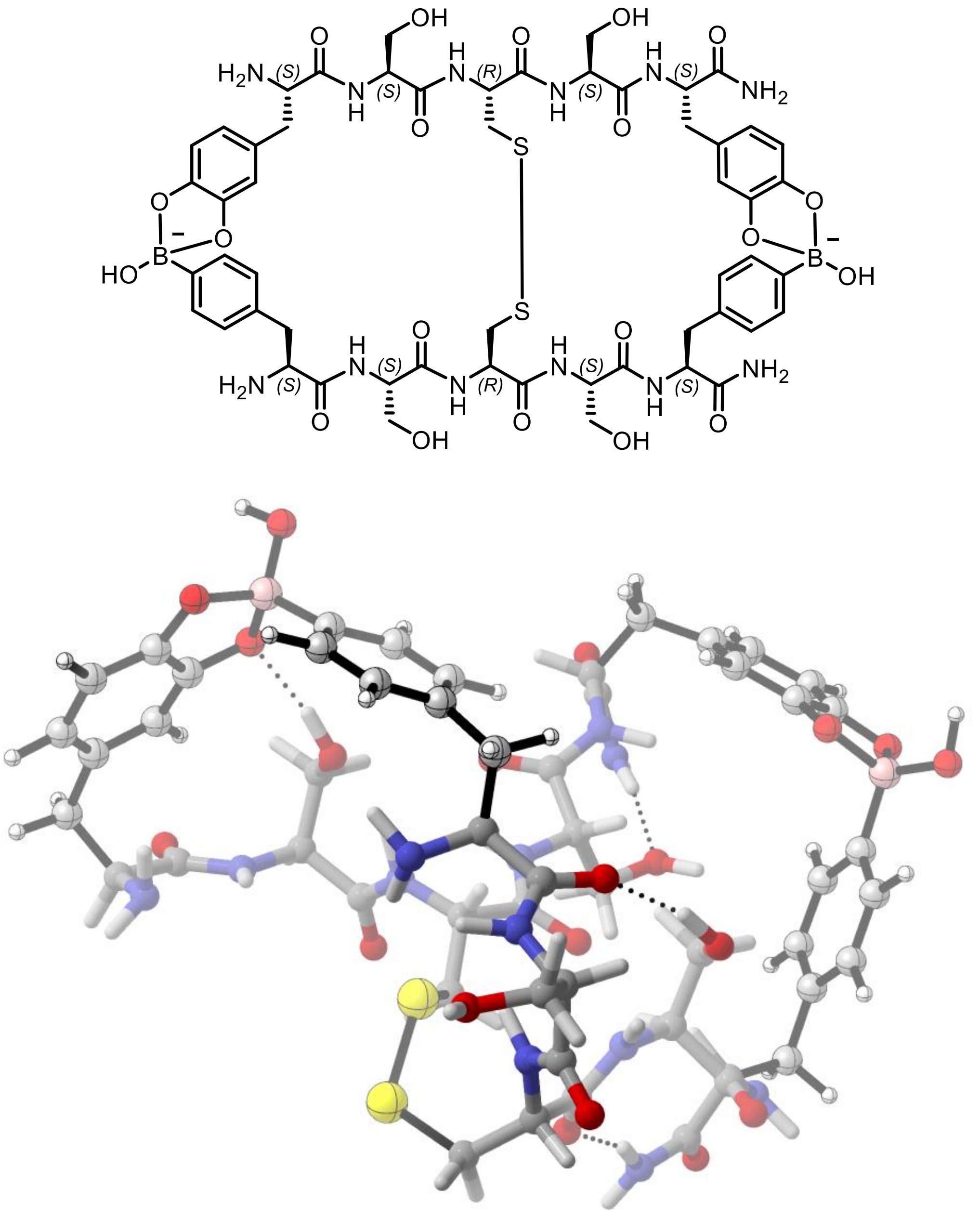

Figure S 30: Structure of the low-lying conformation of 2_{Ox} with a parallel topology (overall charge 2−).

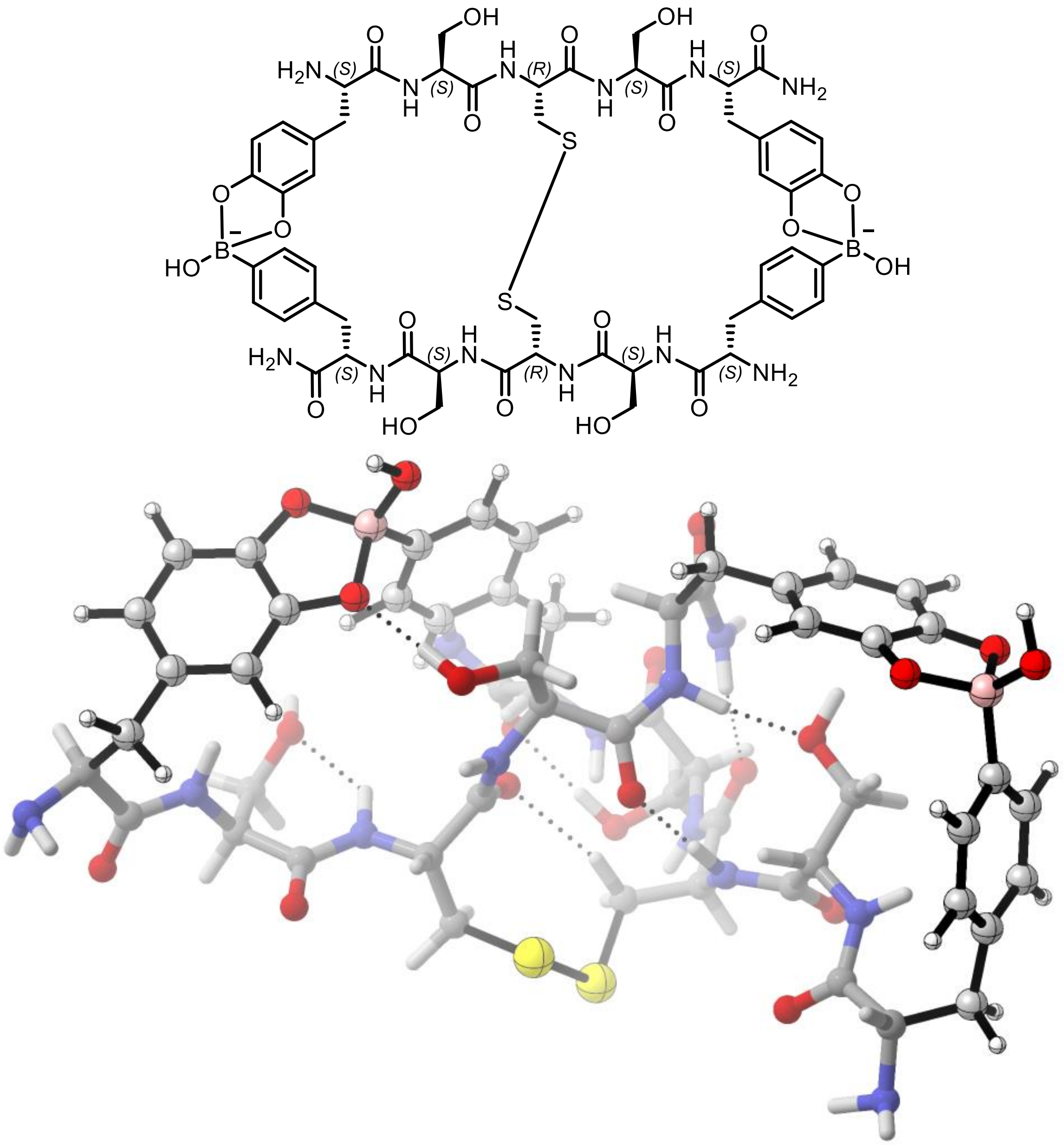

Figure S 31: Structure of the low-lying conformation of 2_{Ox} with an antiparallel topology (overall charge 2−).

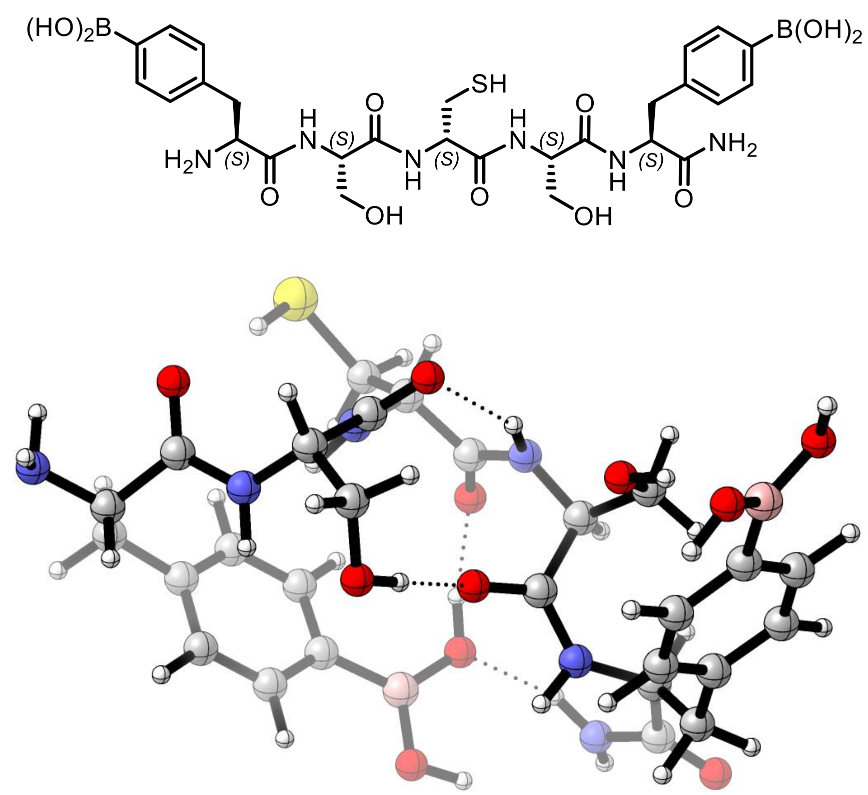

Figure S 32: Skeletal structure and low-lying conformer of BScSB.

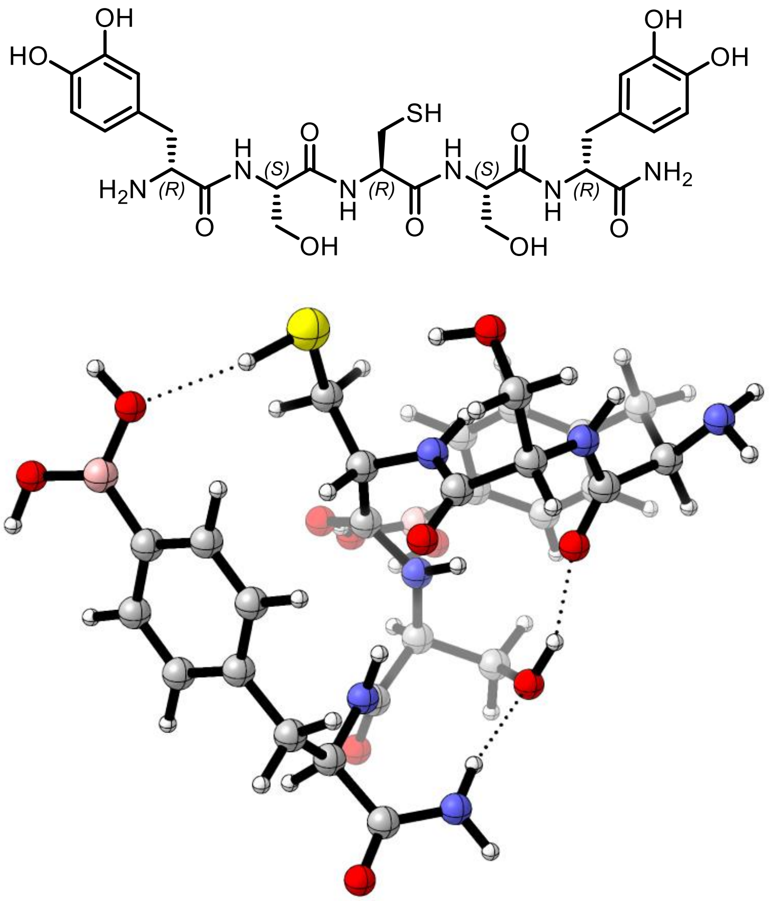

Figure S 33: Skeletal structure and low-lying conformer of bSCSb.

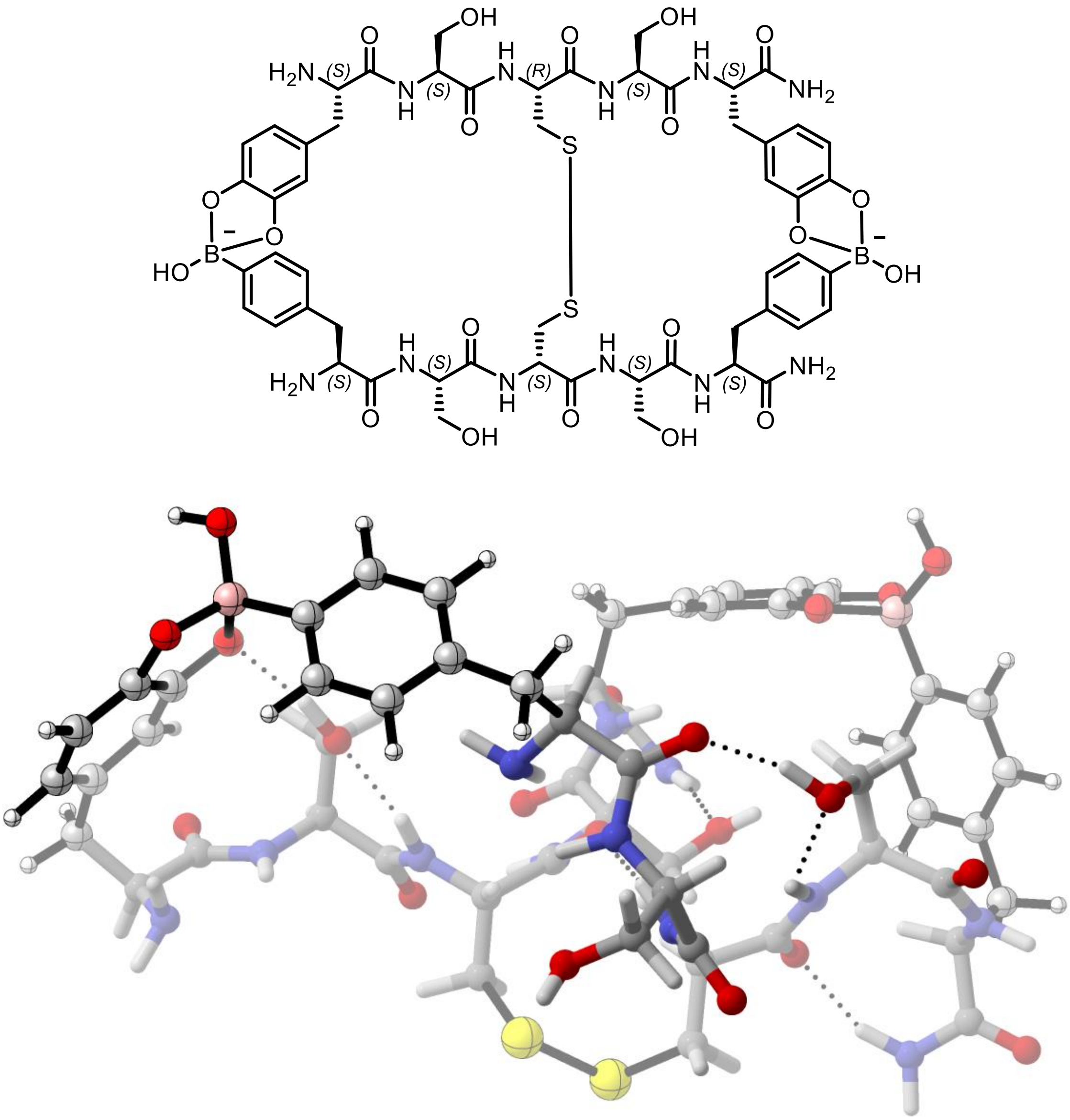

Figure S 34: Structure of BScSB≃OSCSO with a parallel topology (overall charge 2−).

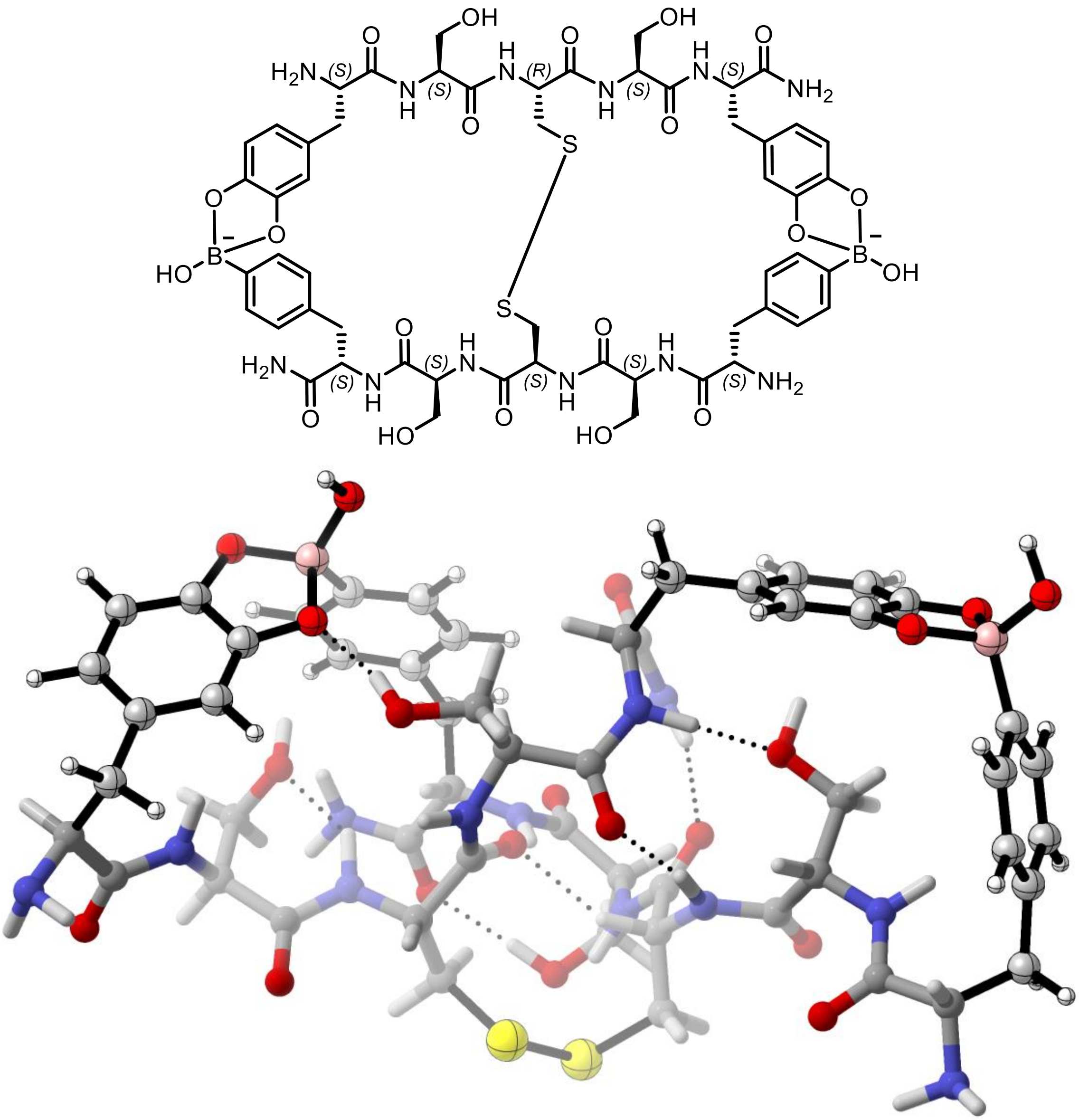

Figure S 35: Structure of BScSB≃OSCSO with an antiparallel topology (overall charge 2−).

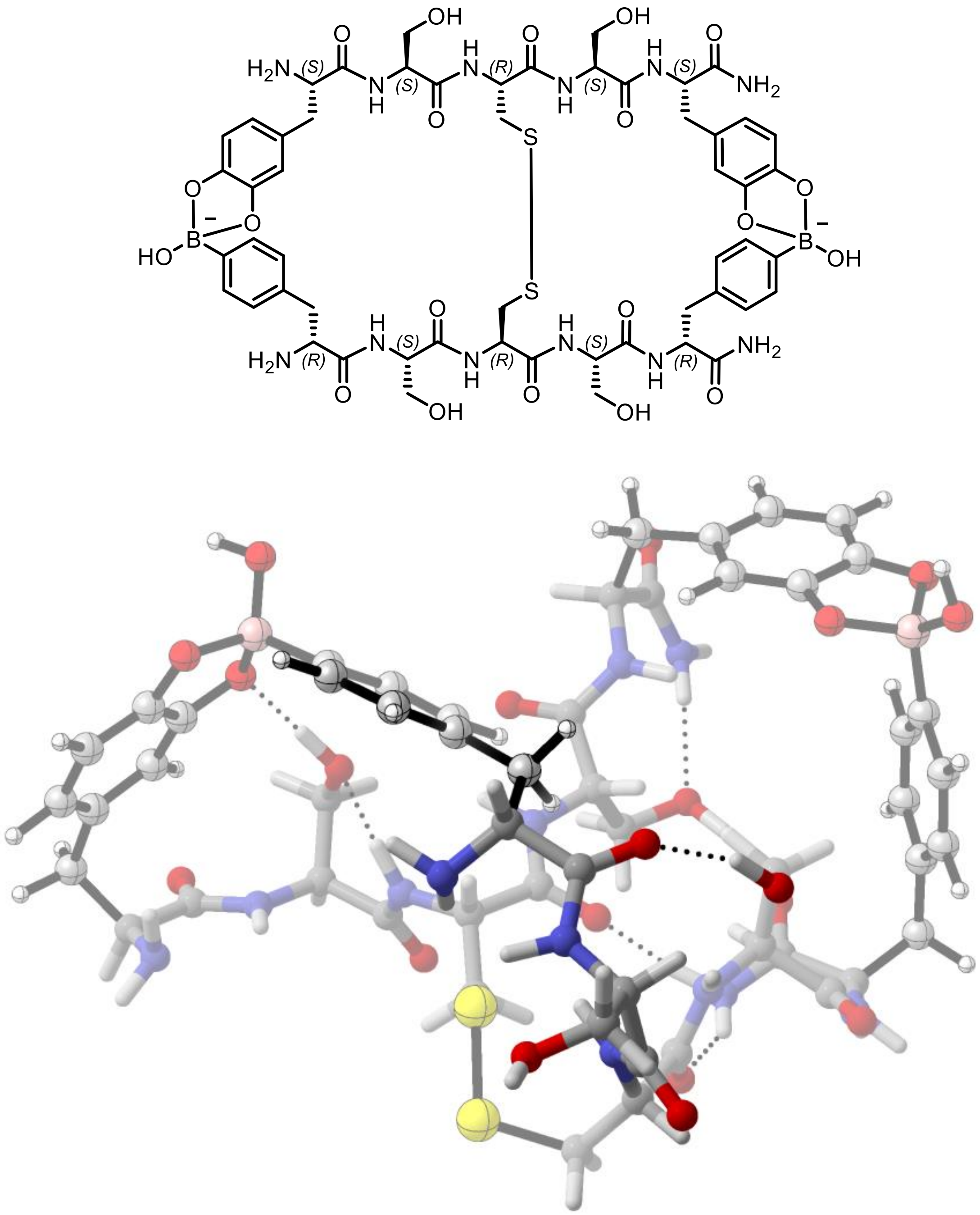

Figure S 36: Structure of bSCSb≃OSCSO with a parallel topology (overall charge 2−).

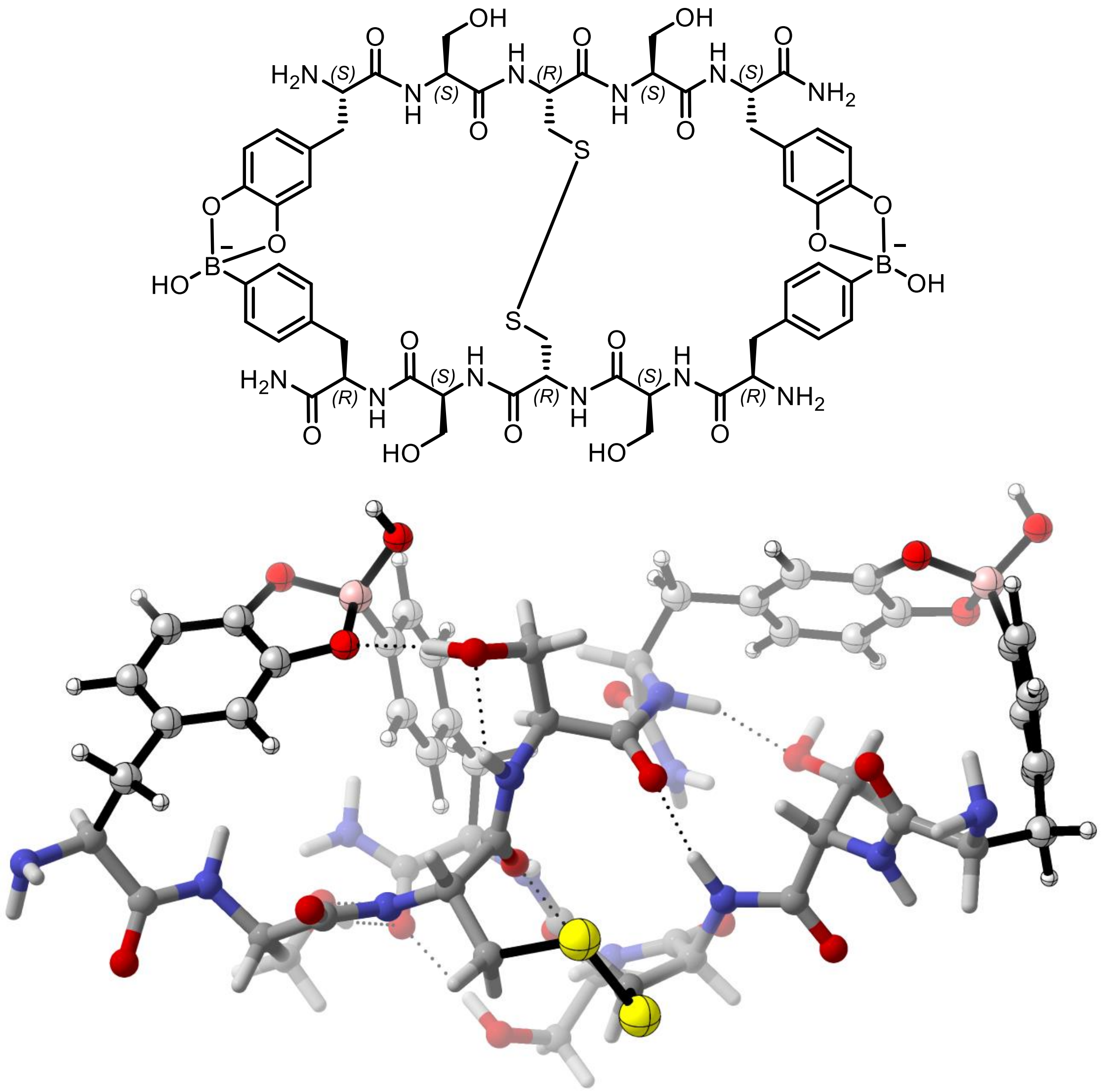

Figure S 37: Structure of bSCSb≃OSCSO with an antiparallel topology (overall charge 2−).

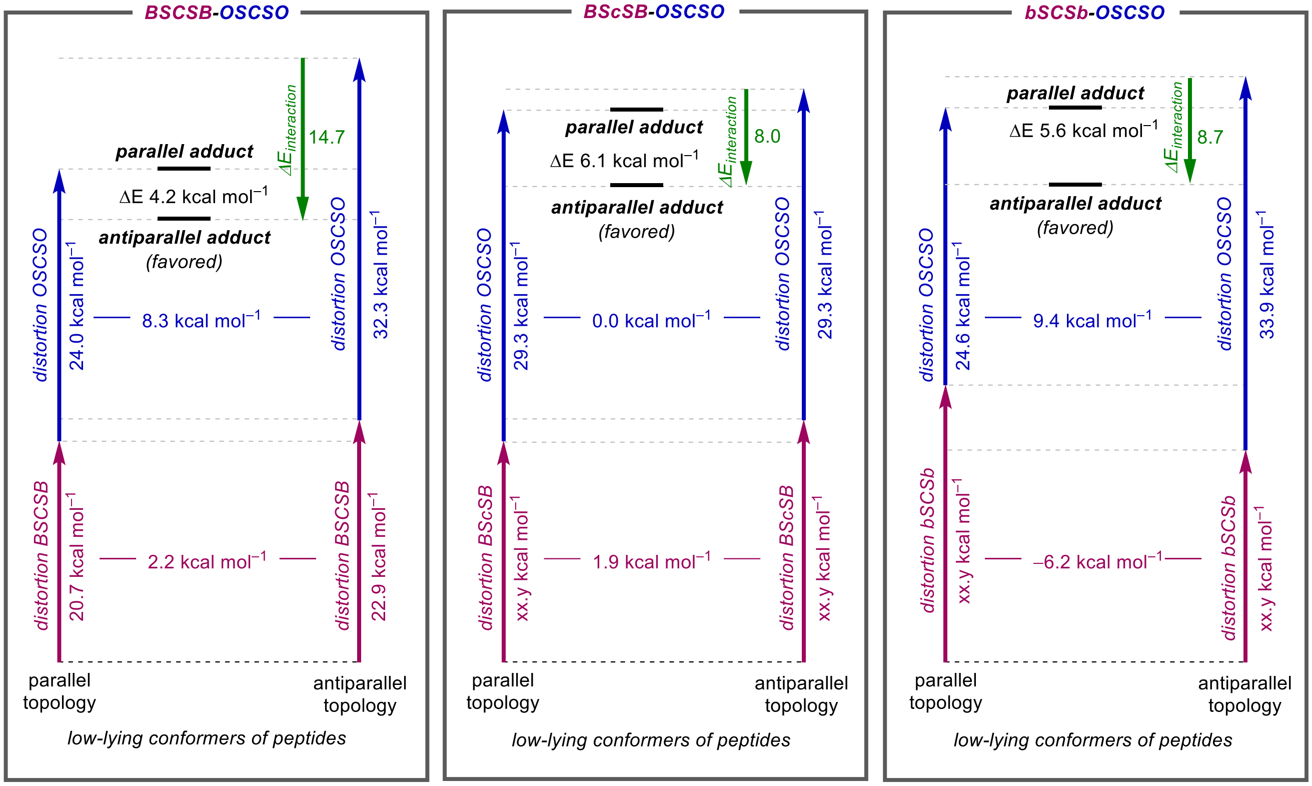

*Figure S 38: Individual distortion/interaction analysis of parallel and antiparallel topologies of BSCSB-OSCSO (**2**$_{Ox}$), BScSB-OSCSO and bSCSb-OSCSO. Antiparallel topology is favored for all three cases.*

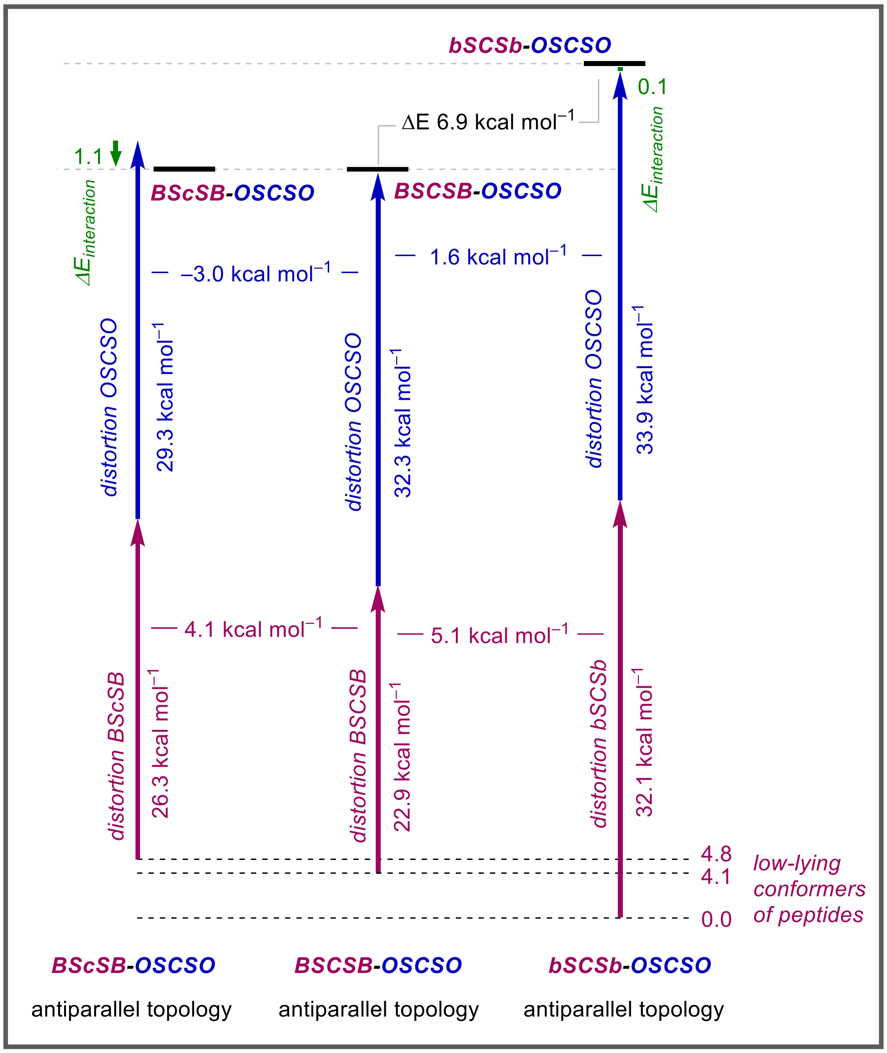

*Figure S 39: Distortion/interaction analysis of antiparallel topologies of BScSB-OSCSO and bSCSb-OSCSO relative to BSCSB-OSCSO (**2**$_{Ox}$). Interaction energies are similar in all three cases. Difference in the overall electronic energies arises from different distortion energies of the peptides.*

2_{Ox} parallel topology

2_{Ox} antiparallel topology (favored)

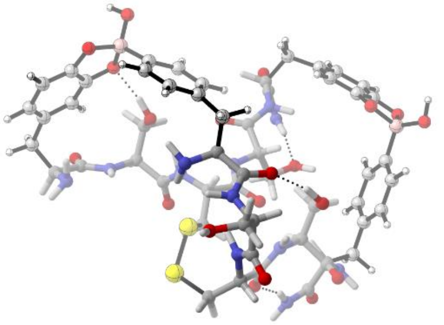

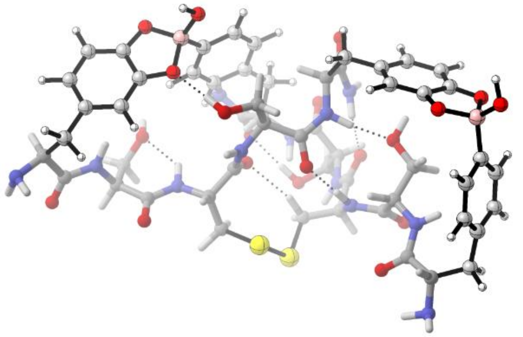

2_{Ox} parallel		2_{Ox} antiparallel
3.5 kcal mol^{-1}	**ΔG**	0.0 kcal mol^{-1}
4.2 kcal mol^{-1}	**ΔE**	0.0 kcal mol^{-1}

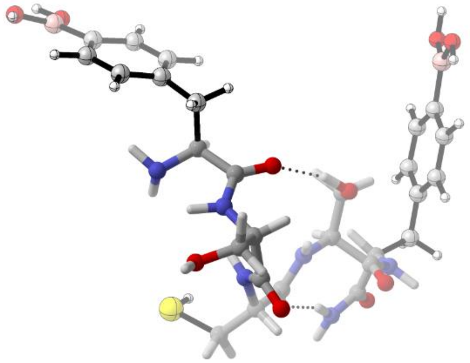

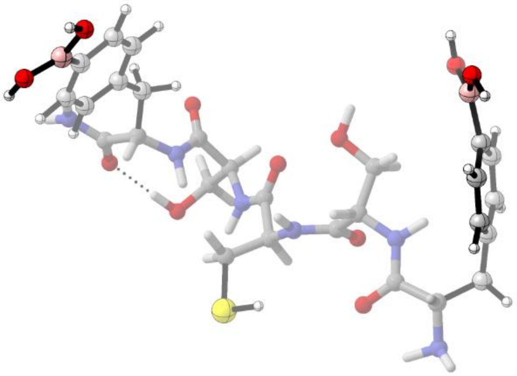

0.0 kcal mol^{-1}	**BSCSB** **$\Delta E_{distortion}$**	2.2 kcal mol^{-1}

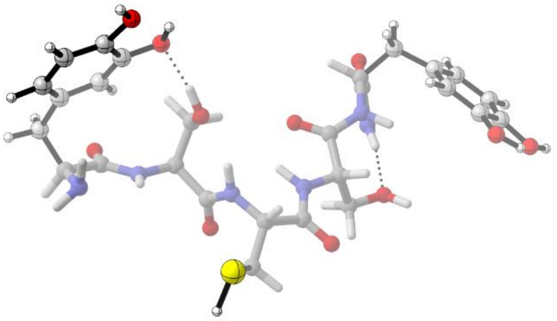

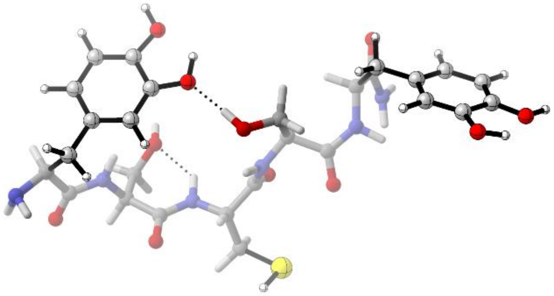

0.0 kcal mol^{-1}	**OSCSO** **$\Delta E_{distortion}$**	8.3 kcal mol^{-1}
14.7 kcal mol^{-1}	**$\Delta E_{interaction}$**	0.0 kcal mol^{-1}

Figure S 40: Distortion/interaction analysis of the low-lying conformations of parallel and antiparallel topologies. Distortion energy of the structures of BSCSB and OSCSO is greater when adopting an antiparallel topology. However, the higher energy of interaction in this topology (as a result of several simultaneous non-covalent interactions), makes this antiparallel topology favored over the parallel topology.

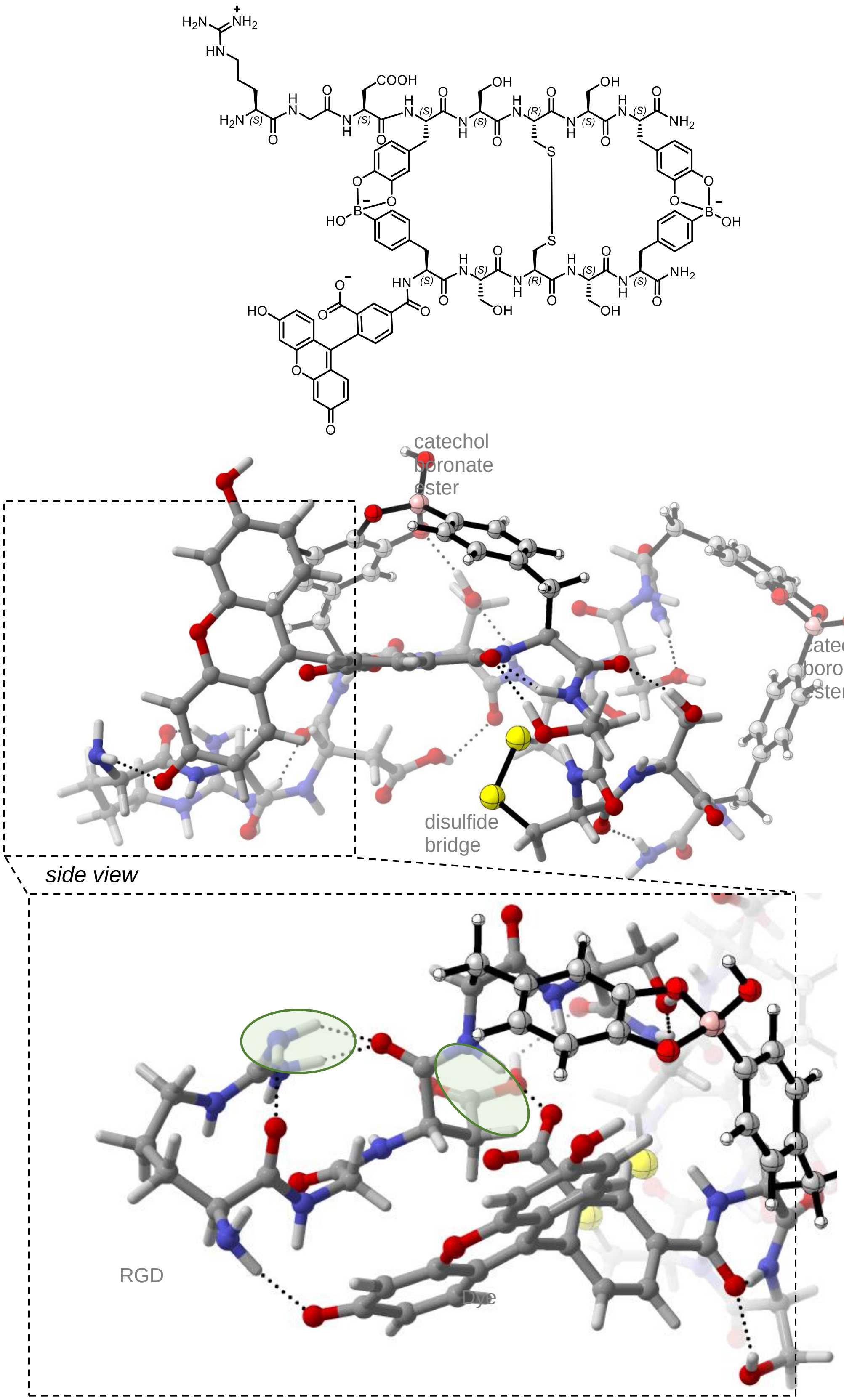

Figure S 41: Structure of the low-lying conformation of fluorescein≃RGD with parallel topology (overall charge 2−).

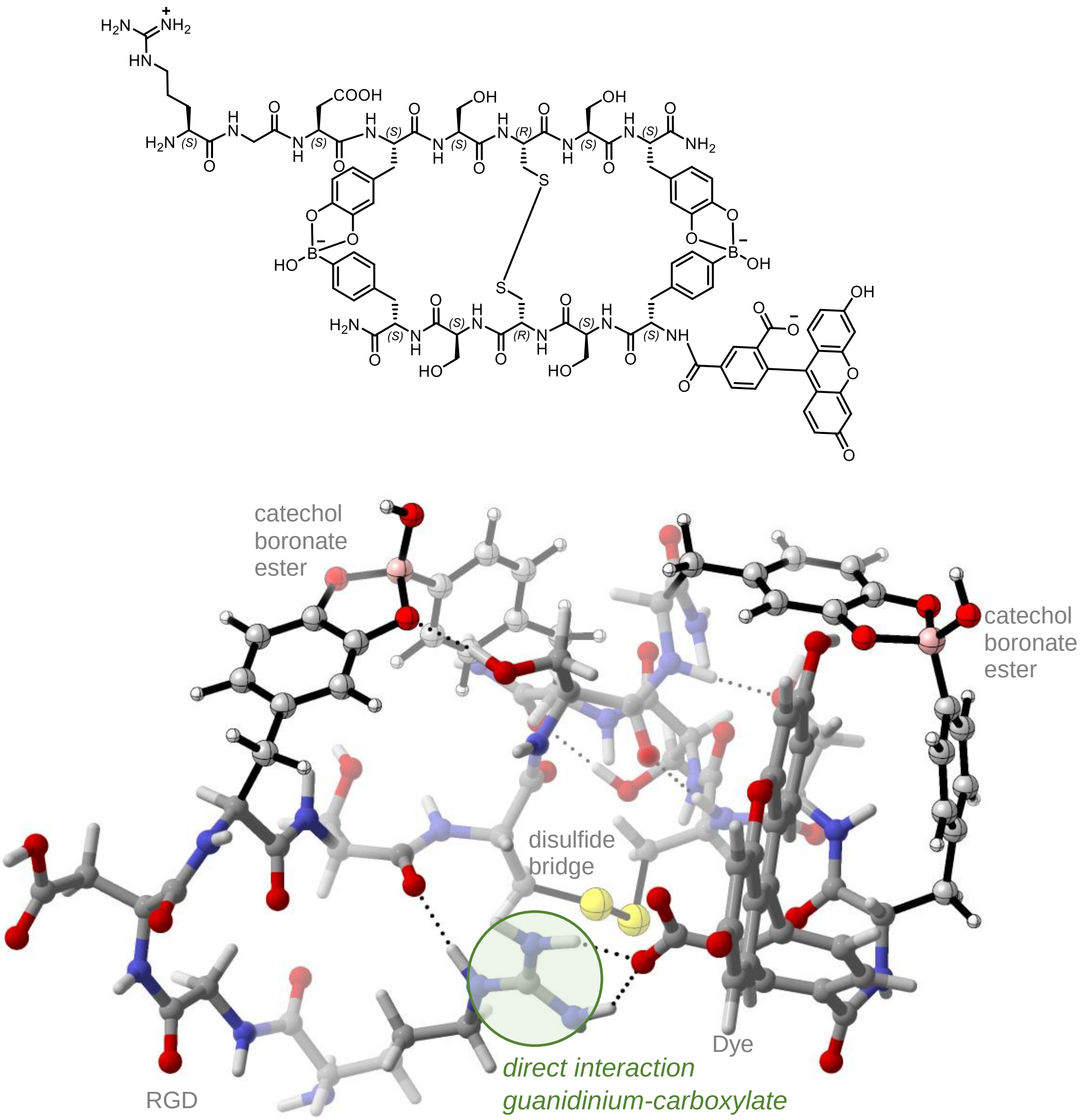

Figure S 42: Structure of fluorescein≃RGD with antiparallel topology (overall charge 2−).

Table S 3: Energies and Cartesian Coordinates.

Calculated data for the optimized structures

Structure	S-S bond distance (Å)	SCF energy (a.u.)	E_{rel} (kcal mol^{-1})		Quasiharmonic free energy correction (a.u.)	qh-G (a.u.)	qh-G_{rel} (kcal mol^{-1})	
**2_{Ox} (BSCSB≃OSCSO) parallel**	2.07	−5106.054384	4.2	4.2	1.132829	−5104.921555	3.5	**3.5**
**2_{Ox} (BSCSB≃OSCSO) antiparallel**	2.08	−5106.061017	0.0	**0.0**	1.133894	−5104.927123	0.0	**0.0**
**BScSB≃OSCSO parallel**	2.09	−5106.051194	6.1	6.2	1.133404	−5104.917791	4.8	**5.9**
**BScSB≃OSCSO antiparallel**	2.10	−5106.060946	0.0	**0.0**	1.135560	−5104.925386	0.0	**1.1**
**bSCSb≃OSCSO parallel**	2.07	−5106.041079	5.6	12.5	1.135425	−5104.905654	4.2	**13.5**
**bSCSb≃OSCSO antiparallel**	2.10	−5106.049949	0.0	**6.9**	1.137538	−5104.912411	0.0	**9.2**
**fluorescein≃RGD (BSCSB≃OSCSO) parallel**	2.07	−7537.884226	3.3		1.720024	−7536.164203	7.5	
**fluorescein≃RGD (BSCSB≃OSCSO) antiparallel**	2.08	−7537.889473	0.0		1.713384	−7536.176089	0.0	
**OSCSO**	N/A	−2604.946718	N/A		0.579664	−2604.367054	N/A	
**BSCSB**	N/A	−2656.115667	4.1		0.611686	−2655.503981	4.6	
**BScSB**	N/A	−2656.114543	4.8		0.609962	−2655.504581	4.2	
**bSCSb**	N/A	−2656.122156	0.0		0.610918	−2655.511239	0.0	

15. Cell uptake studies

Sample preparation protocol. 20 µL of 2 mM solutions of DL488-BSCSB and either OSCSO, OSCSO and TAT or TAT-OSCSO were mixed together to obtain 40 µL of 1 mM solution of preassembled conjugate (in 10 mM phosphate buffer pH = 8 with 10% DMSO). The conjugate was subsequently oxidized by addition of 1 µL 75 mM potassium peroxymonosulfate (Oxone®) solution in 100 mM PB, pH = 7.4 to obtain its final concentration as 1.8 mM. Prepared conjugate was used directly for the cell studies without any purification.

Cell culture. The human cell line A549 from adenocarcinomic lung tissue (American Type Culture Collection, Manassas, VA, US) was cultured in Dulbecco's modified Eagle medium (DMEM 4.5 g/L D-Glucose, L-Glutamine; Gibco by ThermoFisher Scientific, Germany) supplemented with 10% fetal calf serum (FCS; Sigma Aldrich, Germany) and 1% Penicillin/Streptomycin (Invitrogen, Germany) at a humified atmosphere with 37 °C and 5% CO_2 . Fresh culture medium was replaced every two days and cells were subcultured after reaching 80% confluence.

Evaluation of cell uptake. A549 cells were detached by TrypLE Select (Gibco by ThermoFisher Scientific, Germany) and centrifuged 5 min at 1000 rpm. Collected cells were resuspended in DMEM-low glucose (1000 mg/L D-Glucose; Sigma-Aldrich, Germany) supplemented with 10% fetal calf serum (FCS; Sigma Aldrich, Germany) and 1% Penicillin/Streptomycin (Invitrogen, Germany) and seeded in µ-slide 8-well chambers (Ibidi GmbH, Germany) at a cell density of 10000 cells/well. 24 h after seeding, cells were treated with 10 µM of DL488-BSCSB≃OSCSO-TAT, DL488-BSCSB≃OSCSO+TAT or DL488-BSGSB~OSCSO-TAT. Untreated cells were used as control. For the uptake studies in acidic conditions, treatments were applied in DMEM low glucose (Agilent, Germany) without sodium bicarbonate supplemented with 10% fetal calf serum (FCS; Sigma Aldrich, Germany) and 1% Penicillin/Streptomycin (Invitrogen, Germany) at pH 6.4 and cells were incubated at 37 °C in absence of CO_2. After 24 h cells were washed with phosphate buffered saline (PBS) and fixed 15 min with 4% paraformaldehyde. Cell nuclei were stained with 5µg/ml of 4′,6-Diamidino-2-phenylindole dihydrochloride (DAPI; Sigma-Aldrich, Germany) diluted in PBS/0.1% Triton solution. Confocal z-stacks were taken by a TCS SP5 confocal microscope (Leica, Germany) (DAPI: Ex=405 nm, Em= 419-471 nm; DL488: Ex=488 nm, Em=503-537 nm) and orthogonal sections were obtained by image processing using Fiji (ImageJ). Experiment was carried out in triplicates from three independent cultures.

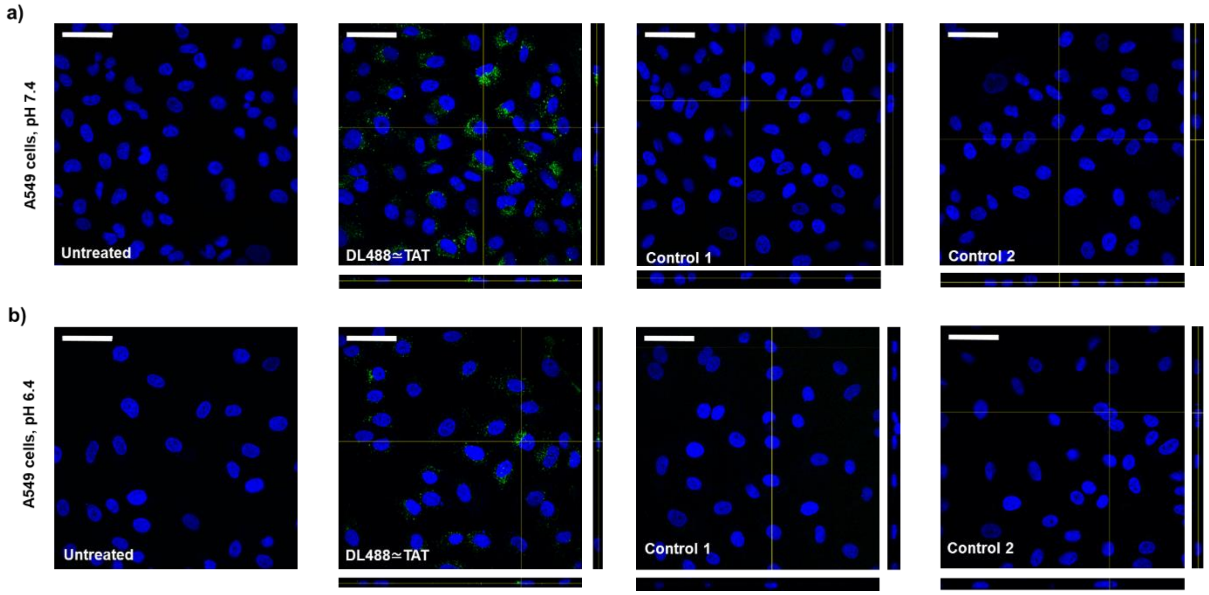

Figure S 43: Confocal images with orthogonal views to investigate cell uptake of bis-peptides, controls (10 µM) and untreated cells at a) physiological pH or b) acidic pH. Control 1: ***DL488-BSCSB≃OSCSO + TAT*** *incubated with free TAT sequence; Control 2: a conjugate without disulfide bridge* ***DL488-BSGSB~OSCSO-TAT*** *where cysteine was replaced with glycine. Scale bar = 50 µm*

References

1. Schrödinger Release 2018-4: Maestro 11.8, Schrödinger, LLC, New York, NY, 2018.
2. Roos, K.; Wu, C.; Damm, W.; Reboul, M.; Stevenson, J. M.; Lu, C.; Dahlgren, M. K.; Mondal, S.; Chen, W.; Wang, L.; Abel, R.; Friesner, R. A.; Harder, E. D., OPLS3e: Extending Force Field Coverage for Drug-Like Small Molecules. *Journal of Chemical Theory and Computation* **2019,** *15* (3), 1863-1874.
3. Frisch, M. J.; Trucks, G. W.; Schlegel, H. B.; Scuseria, G. E.; Robb, M. A.; Cheeseman, J. R.; Scalmani, G.; Barone, V.; Mennucci, B.; Petersson, G. A.; Nakatsuji, H.; Caricato, M.; Li, X.; Hratchian, H. P.; Izmaylov, A. F.; Bloino, J.; Zheng, G.; Sonnenberg, J. L.; Hada, M.; Ehara, M.; Toyota, K.; Fukuda, R.; Hasegawa, J.; Ishida, M.; Nakajima, T.; Honda, Y.; Kitao, O.; Nakai, H.; Vreven, T.; Jr., J. A. M.; Peralta, J. E.; Ogliaro, F.; Bearpark, M.; Heyd, J. J.; Brothers, E.; Kudin, K. N.; Staroverov, V. N.; Kobayashi, R.; Normand, J.; Raghavachari, K.; Rendell, A.; Burant, J. C.; Iyengar, S. S.; Tomasi, J.; Cossi, M.; Rega, N.; Millam, J. M.; Klene, M.; Knox, J. E.; Cross, J. B.; Bakken, V.; Adamo, C.; Jaramillo, J.; Gomperts, R.; Stratmann, R. E.; Yazyev, O.; Austin, A. J.; Cammi, R.; Pomelli, C.; Ochterski, J. W.; Martin, R. L.; Morokuma, K.; Zakrzewski, V. G.; Voth, G. A.; Salvador, P.; Dannenberg, J. J.; Dapprich, S.; Daniels, A. D.; Ö. Farkas; Foresman, J. B.; Ortiz, J. V.; Cioslowski, J.; Fox, D. J. GAUSSIAN 09 (Revision D.01), Gaussian, Inc.: Wallingford CT, 2009.
4. CYLview20; Legault, C. Y., Université de Sherbrooke, 2020 (http://www.cylview.org)
5. Zhao, Y.; Truhlar, D. G., The M06 suite of density functionals for main group thermochemistry, thermochemical kinetics, noncovalent interactions, excited states, and transition elements: two new functionals and systematic testing of four M06-class functionals and 12 other functionals. *Theoretical Chemistry Accounts* **2008,** *120* (1), 215-241.

6. Cances, E.; Mennucci, B.; Tomasi, J., A new integral equation formalism for the polarizable continuum model: Theoretical background and applications to isotropic and anisotropic dielectrics. *J Chem Phys* **1997,** *107* (8), 3032-3041.
7. Cossi, M.; Barone, V.; Mennucci, B.; Tomasi, J., Ab initio study of ionic solutions by a polarizable continuum dielectric model. *Chem Phys Lett* **1998,** *286* (3-4), 253-260.
8. Mennucci, B.; Tomasi, J., Continuum solvation models: A new approach to the problem of solute's charge distribution and cavity boundaries. *J Chem Phys* **1997,** *106* (12), 5151-5158.
9. Tomasi, J.; Mennucci, B.; Cammi, R., Quantum mechanical continuum solvation models. *Chem Rev* **2005,** *105* (8), 2999-3093.
10. Marenich, A. V.; Cramer, C. J.; Truhlar, D. G., Universal Solvation Model Based on Solute Electron Density and on a Continuum Model of the Solvent Defined by the Bulk Dielectric Constant and Atomic Surface Tensions. *J Phys Chem B* **2009,** *113* (18), 6378-6396.
11. Ribeiro, R. F.; Marenich, A. V.; Cramer, C. J.; Truhlar, D. G., Use of Solution-Phase Vibrational Frequencies in Continuum Models for the Free Energy of Solvation. *The Journal of Physical Chemistry B* **2011,** *115* (49), 14556-14562.
12. Marco Hebel, Andreas Riegger, Maksymilian M. Zegota, Gönül Kizilsavas, Jasmina Gačanin, Michaela Pieszka, Thorsten Lückerath, Jaime A. S. Coelho, Manfred Wagner, Pedro M. P. GoisPedro M. P. Gois, David Y. W. Ng*, and Tanja Weil*, Sequence Programming with Dynamic Boronic Acid/Catechol Binary Codes. J. Am. Chem. Soc. 2019, 141, 36, 14026–14031
13. Stefan P. A. Hinkes and Christian D. P. Klein*, Virtues of Volatility: A Facile Transesterification Approach to Boronic Acids. Org. Lett. 2019, 21, 9, 3048–3052
